# Chronic HBV Infection Disrupts CCL5‐Secreting cNK Cells and Attenuates Liver Accumulation and Activation of DCs and HBV‐Specific T Cells

**DOI:** 10.1002/advs.76696

**Published:** 2026-07-20

**Authors:** Ailu Yang, Yucan Wang, Yating Yu, Jing Wu, Cuiping Bao, Zixuan Wang, Qiuju Han, Zhigang Tian, Jian Zhang, Huajun Zhao

**Affiliations:** ^1^ State Key Laboratory of Discovery and Utilization of Functional Components in Traditional Chinese Medicine School of Pharmaceutical Sciences Shandong University Jinan Shandong China; ^2^ School of Life Sciences University of Science and Technology of China Hefei China

**Keywords:** CD8^+^ T‐cell, chronic hepatitis B, dendritic cells, NK cell‐DC axis, NK cells

## Abstract

Understanding the immunological mechanisms underlying chronic hepatitis B virus (HBV) infection‐induced immune tolerance is critical for developing effective therapeutic strategies, yet the mechanisms by which natural killer (NK)‐cell subsets regulate dendritic cell (DC)‐mediated antiviral immunity during chronic HBV infection remain unclear. Here, we demonstrated that chronic HBV infection disrupted intrahepatic DCs accumulation and activation by suppressing CC chemokine ligand 5 (CCL5) production from hepatic NK cells. Importantly, chronic HBV infection reduced the number of hepatic conventional NK (cNK) cells, the main source of hepatic CCL5, whereas liver‐resident NK (LrNK) cells increased and exhibited lower CCL5 expression and immunosuppressive features. Adoptive transfer of CCL5‐competent hepatic cNK cells promoted DCs accumulation and activation, enhanced HBV‐specific CD8^+^ T‐cell responses, and facilitated viral clearance. Mechanistically, chronic HBV infection promoted the activation of adenosine A2a receptor (ADORA2A)/nuclear factor kappa‐B (NF‐κB) pathway in hepatic NK cells, thereby antagonizing NF‐κB activation, reducing the proportion of cNK cells, suppressing CCL5 production, and ultimately impairing hepatic aggregation and activation of DCs, leading to defective anti‐HBV immune responses. This study revealed a novel mechanism involved in chronic HBV infection‐induced immune tolerance and indicated that targeting the ADORA2A/NFκB/CCL5 axis in cNK cells may represent a potential therapeutic strategy for chronic HBV infection.

## Graphical abstract

ADORA2A activation suppresses NFκB‐dependent CCL5 production in cNK cells during chronic HBV infection, disrupting intrahepatic DC recruitment and HBV‐specific CD8^+^ T‐cell differentiation and function. Targeting the ADORA2A/NFκB/CCL5 axis restores antiviral immunity and facilitates HBV clearance.

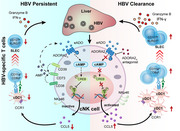

## 1 Introduction

Chronic hepatitis B virus (HBV) infection is a global health problem and a major risk factor for the development of liver fibrosis, cirrhosis, and hepatocellular carcinoma [1]. Chronic hepatitis B (CHB) develops due to the complex interactions between HBV and the host immune system, which results in the dysfunction of dendritic cells (DCs), natural killer (NK) cells, and CD8^+^ T cells [2, 3, 4, 5], contributing to liver immune tolerance. Therefore, exploration of the new immunological mechanisms involved in chronic HBV infection‐induced immune tolerance is important for the development of new therapeutic strategies to achieve long‐term virologic control.

Antigen‐specific CD8^+^ T cells are important in immune surveillance and defense against infections. However, HBV‐specific CD8^+^ T cells often exhibit an exhausted phenotype in CHB patients [6, 7], resulting in an impaired ability to eliminate HBV. Naïve CD8^+^ T cells can differentiate into terminally differentiated short‐lived effector cells (SLECs) and memory precursor effector cells (MPECs) upon pathogen stimulation [8]. Compared with MPECs, SLECs are the dominant effector and memory cells for HBV elimination and against HBV recurrence [9]. Multiple attempts have been made to restore the effector function of CD8^+^ T cells, particularly by inducing SLECs, to achieve HBV clearance [2, 9, 10, 11]. As specialized antigen‐presenting cells, DCs determine the activation and differentiation of T cells [12]. Classical dendritic cells (cDCs) include two main subsets: conventional type 1 DCs (cDC1s) and conventional type 2 DCs (cDC2s). cDC1s can be further divided into CD8α^+^ and CD103^+^ cDC1s, which play important roles in antigen cross‐presentation and CD8^+^ T‐cell activation. cDC2s elicit mainly Th2‐mediated immune responses [13]. cDC1s migrate to the inflammatory site via chemokine‐mediated recruitment, thereby mediating the infiltration and activation of T cells [14]. Our studies and others have shown that a high viral load leads to the dysfunction of cDCs and T cells, contributing to persistent HBV infection and progressive liver disease [3, 4, 15].

NK cells are the main type of innate lymphocytes and can directly regulate T‐cell responses against pathogens in an IFN‐γ secretion‐ and PD‐L1‐dependent manner [16, 17]. Recent studies have also identified an integral role for NK cells in promoting DC maturation and recruitment to tumors via various chemokines, such as XCL1, XCL2, CCL5, and growth factor Fms‐like tyrosine kinase 3 ligand (FLT3L), in turn promoting the infiltration and activation of T cells [18, 19, 20]. However, a high viral load disrupts NK cell functions in CHB patients [21, 22], contributing to the progression of CHB and hepatocellular carcinoma (HCC) [5]. However, the regulatory effect of the NK cell‐DC interaction on the CD8^+^ T‐cell response and progressive liver disease in CHB remains unclear.

In this study, we used HBV‐carrier mice and published single‐cell RNA sequencing (scRNA‐seq) datasets from healthy donors (HDs) and immune‐tolerant CHB patients to explore the interaction among NK cells, DCs, and CD8^+^ T cells. We revealed the NK cell subsets involved in DC‐mediated regulatory effects on the differentiation and function of CD8^+^ T cells in chronic HBV infection. We found that chronic HBV infection decreased the number of NK cells and suppressed CCL5 production by hepatic cNK cells via the ADORA2A/NFκB signaling pathway, which impaired hepatic accumulation and activation of DCs, impeding anti‐HBV response. Strategies that target the ADORA2A/NFκB/CCL5 signaling axis in NK cells might be potential new treatments for chronic HBV infection.

## 2 Material and Methods

### 2.1 Animals and Reagents

Male C57BL/6J mice (5 weeks old) were purchased from Beijing HFK Bioscience Co., Ltd. (Beijing, China). CCL5‐knockout (CCL5‐KO) mice were purchased from Shanghai Model Organisms Center, Inc. (Shanghai, China). The recombinant adeno‐associated virus (rAAV)8‐HBV1.3 vectors containing 1.3 copies of the HBV genome (genotype D, subtype ayw) were purchased from PackGene Biotech (Guangzhou, China). The HBV carrier mouse model was established via intravenous injection of 1 × 10^10^ vg rAAV8‐HBV1.3 vector, as previously described [23]. The characteristics of this mouse model could well mimic the tolerant state of human chronic HBV carriers. Six weeks later, serum HBsAg was detected at week 6 after the injection, and the mice with serum HBsAg levels > 500 ng/mL were defined as HBV‐carrier mice. All animal experiments were performed in accordance with the Guidelines for the Care and Use of Laboratory Animals and the Ethical Committee of Shandong University and were approved by the Institutional Animal Care and Use Committee of Shandong University (approval number: 240142).

### 2.2 NK Cell Sorting, Transfer and CD8^+^ T Cell Depletion

Hepatic NK cells from WT or CCL5 knockout (CCL5‐KO) mice were sorted with the MojoSort mouse NK cell isolation kit (#480050, Biolegend). Hepatic LrNK cells (CD3^−^ CD19^−^ NK1.1^+^ CD49a^+^ DX5^−^) and cNK cells (CD3^−^ CD19^−^ NK1.1^+^ CD49a^−^ DX5^+^) from WT mice were sorted with a Beckman Coulter MoFlo Astrios EQ cell sorter. The purity of these sorted cell populations was verified by flow cytometry (>90%). To analyze the regulatory role of NK cells in the anti‐HBV immune response, these purified NK cell subsets were transferred into new recipient HBV‐carrier mice in which NK cells were depleted by anti‐NK1.1. For CD8^+^ T cell depletion, the mice were injected intraperitoneally (i.p.) with 200 µg of anti‐CD8α (#A2102, Selleck) once a day for three consecutive days before adoptive transfer of NK cells.

### 2.3 NK Cell Depletion In Vivo

For NK cell depletion, the mice were injected intraperitoneally (i.p.) with 100 µL of anti‐NK1.1 (PK136, 200 µg/mouse) once a day for three consecutive days before adoptive transfer of NK cells, and the efficacy of NK cell depletion was confirmed by flow cytometry.

### 2.4 Mononuclear Cell Isolation

Isolation of liver mononuclear cells (MNC) and splenocytes was performed as previously described [9, 15]. Liver tissues excised from the mice were shredded and then were passed through a 200‐µm nylon cell strainer, followed by centrifugation at 100 × g for 1 min to remove the hepatocytes. The single‐cell suspension was then centrifuged at 400 × g for 10 min, and the cell precipitates were suspended in 40% Percoll (Cytiva, USA), followed by centrifugation at 800 × g for 25 min. Hepatic mononuclear cells (MNCs) were obtained after red blood cell lysis and washing with 1× PBS. The spleen samples were passed through a 200‐µm nylon cell strainer, and splenic MNCs were obtained after red blood cell lysis and washing with 1× PBS. The draining lymph node (dLN) samples were passed through a 200‐µm nylon cell strainer, and dLN MNCs were obtained after washing with 1× PBS.

### 2.5 Antibody Staining and Flow Cytometry

Single‐cell suspensions were preincubated with Fc‐receptor blocking solution (anti‐mouse CD16/32, eBioscience, California, USA) and stained with the indicated mAb conjugates at 4°C for at least 1 h. For analysis of intracellular molecules, the cells were fixed and permeabilized with fixation and permeabilization buffers (eBioscience, California, USA) according to the manufacturer's instructions. Data were collected via a BD FACSymphony A3 or BD FACSCelesta system (BD Biosciences) and analyzed with FlowJo V10 software (FlowJo, LLC, Ashland, OR, USA). The antibodies used are listed in Supplementary Table S1.

### 2.6 Bone Marrow‐Derived Dendritic Cells (BMDCs)

Murine BMDCs were generated as previously described [9, 15]. Briefly, murine bone marrow MNCs were cultured in RPMI 1640 medium supplemented with 5 ng/mL rmIL‐4 (*E. coli*, Peprotech, Rocky Hill, USA) and 10 ng/mL rmGM‐CSF (*E. coli*, Peprotech, Rocky Hill, USA), and two‐thirds of the medium was replaced on Days 3 and 5. These cells were harvested on Day 7, and the percentage of CD11c^+^ cells was analyzed via flow cytometry to confirm the purity of the BMDCs (>90%). For the generation of bone marrow‐derived CD103^+^ DCs, murine bone marrow MNCs were cultured in RPMI 1640 medium supplemented with 160 ng/mL rmFLT3L (*E. coli*, Peprotech, Rocky Hill, USA) and 25 ng/mL rmGM‐CSF (*E. coli*, Peprotech, Rocky Hill, USA), and CD11c^+^ CD103^+^ cells were identified as CD103^+^ DCs.

### 2.7 DC Migration Assay

The chemotactic effects of NK cells or CCL5 on DCs were measured via 24‐well transwell chambers with gelatin‐coated polycarbonate membrane filters (Corning Costar, USA) [24]. Briefly, 1 × 10^6^ BMDCs were added to the upper chamber in 100 µL of medium (1% FBS). The lower chamber was subsequently filled with 600 µL of medium (10% FBS) containing 10 or 100 ng/mL CCL5 (Biolegend, San Diego, USA), CXCL2 (Abclonal, Wuhan, China), or 2 × 10^5^ NK cells, and the medium alone group was used as the control. After incubation for 5 h (cytokine chemotaxis assay) or 15 h (NK cell chemotaxis assay), the CD11c^+^ cells that migrated to the lower chamber were harvested and quantified via flow cytometry.

### 2.8 Dataset Analysis

For Gene Ontology enrichment analysis, we used a previously published dataset from the Immunological Genome Project (ImmGen, GSE15907) to analyze the chemokine profiles of different immune cells. And, we used a previously published single‐cell RNA sequencing (scRNAseq) dataset (GSE182159) to analyze the characteristics of different intrahepatic immune cells obtained through ultrasound‐guided percutaneous liver biopsy from HDs, and immune‐tolerant (IT) CHB patients. Patient information from dataset (GSE182159), including virological characteristics, as follows: HDs, age between 33 and 66, without HBsAg level or HBV DNA level, ALT (U/L) between 11 and 31, AST (U/L) between 15 and 27; and immune‐tolerant CHB patients, age between 21 and 55, HBsAg level (IU/mL) between 5652 and 69050, HBV DNA level (log_10_ IU/mL) between 8 and 8.88, ALT (U/L) between 20 and 45.7, AST (U/L) between 19 and 42, as previously reported [25].

### 2.9 Statistical Analysis

Statistical analyses were performed via GraphPad Prism software (v6.0; GraphPad Software, La Jolla, CA, USA). An unpaired Student's *t* test was used for comparisons between two groups, and one‐way ANOVA was used for comparisons among multiple groups. A *p* value less than 0.05 (^*^ *p* < 0.05, ^**^ *p* < 0.01, ^***^ *p* < 0.001, ^****^ *p* < 0.0001) was considered to indicate statistical significance. The data represent the means ± SEMs.

## 3 Results

### 3.1 Chronic HBV Infection Impairs the Function and Intrahepatic Accumulation of DCs

Although our studies and others have shown that chronic HBV infection leads to a decrease in the number and dysfunction of total cDCs [3, 4, 15], the phenotypes and functions of cDC subsets during chronic HBV infection remain unclear. Compared with WT mice, we observed that the proportion and absolute number of intrahepatic DCs, cDC1, and cDC2 subsets, were significantly decreased in HBV‐carrier mice (Figure S1A,B). Meanwhile, chronic HBV infection reduced the expression of CD86, MHC‐I, CD80, and MHC‐II on DCs, cDC1, and cDC2 subsets (Figure S1C). These results suggest that chronic HBV infection disturbs the function and intrahepatic accumulation of DCs.

### 3.2 Chronic HBV Infection Impedes Hepatic Accumulation of DCs via Suppressing the Production of CCL5 by Hepatic NK Cells

**FIGURE 1:**
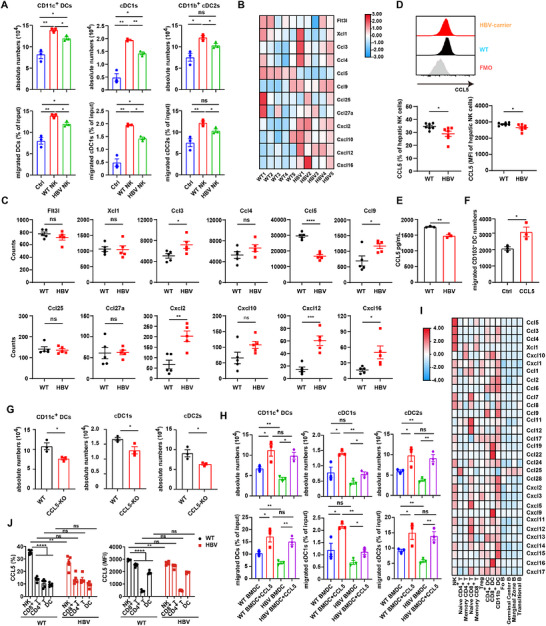
Chronic HBV infection suppresses the production of CCL5 by hepatic NK cells. (A) BMDCs from WT mice were placed in the upper chamber, and hepatic NK cells sorted from WT and HBV‐carrier mice were placed in the lower chamber. After 6 h, the cells that migrated into the lower chamber were collected, and the number (upper) and proportions (lower) of DCs and the cDC1 and cDC2 subsets were detected via flow cytometry. (B,C) NK cells were harvested from the livers of WT mice and HBV‐carrier mice, and then RNA‐seq was performed. Landscape heatmap (B) and statistical plot (C) showed the expression of chemokines in hepatic NK cells from WT and HBV‐carrier mice. (D) Flow cytometric analysis of the expression of CCL5 in hepatic NK cells. (E) The levels of CCL5 secreted by hepatic NK cells were analyzed via ELISAs. (F) CD103^+^ BMDCs derived from WT mice were placed in the upper chamber, and 100 ng/mL recombinant CCL5 was added to the lower chamber. After 6 h, the cells that migrated into the lower chamber were collected, and the numbers of CD103^+^ DCs were determined via flow cytometry. (G) Hepatic NK cells were sorted from WT and CCL5‐KO mice via the MojoSort Mouse NK Cell Isolation Kit. Then, BMDCs from WT mice were placed in the upper chamber, and WT NK or CCL5‐KO NK cells were placed in the lower chamber. After 18 h, the cells that migrated into the lower chamber were collected, and the numbers and proportions of DCs, the cDC1 and cDC2 subsets were determined via flow cytometry. (H) BMDCs from WT mice and HBV‐carrier mice were placed in the upper chamber, and 100 ng/mL recombinant CCL5 was added to the lower chamber. After 5 h, the cells that migrated into the lower chamber were collected, and the number (upper) and proportions (lower) of DCs, the cDC1 and cDC2 subsets were determined via flow cytometry. (I) Heatmap showed the expression levels of chemokines in several immune cells. The data were obtained from the Immunological Genome Project (ImmGen, GSE15907). (J) The expression levels of CCL5 in hepatic NK cells, CD8^+^ T cells, CD4^+^ T cells, and DCs from WT and HBV‐carrier mice were determined via flow cytometry. The data are presented as the means ± SEMs (n ≥ 3). ns, not significant. ^*^ *p* < 0.05, ^**^ *p* < 0.01, ^***^ *p* < 0.001, ^****^ *p* < 0.0001.

NK cells can drive cDC1 accumulation in tumors via the chemoattractants CCL5, XCL1, and FLT3L [18, 19]. However, compared with WT mice, HBV‐carrier mice exhibited impaired activation and maturation of hepatic NK cells, characterized as the decreased expressions of activating receptors NKG2D and NKp46, CD107a, Granzyme B, IFN‐γ, and Ki‐67 (Figure S2). To determine whether the impairment of NK cells contributes to the reduction in hepatic DCs, we used transwell migration assays. As shown in Figure 1A, hepatic NK cells from HBV‐carrier mice had weaker chemotactic effects on bone marrow‐derived DCs (BMDCs) than did NK cells from WT mice (Figure S3A,B). RNA‐seq analysis revealed that chronic HBV infection significantly altered the chemokine profile of hepatic NK cells. Although many chemokines such as CCL9, and CXCL2 slightly up‐regulated in hepatic NK cells from HBV‐carrier mice, CCL5 was the most abundant chemokine and showed dramatically decrease (Figure 1B,C). Flow cytometric analysis and enzyme‐linked immunosorbent assay (ELISA) further confirmed that chronic HBV infection inhibited CCL5 production by hepatic NK cells (Figure 1D,E). Compared with CXCL2, the addition of CCL5 better facilitated the migration of BMDCs from WT and HBV‐carrier mice, indicating the CCL5 played a key role in recruiting DCs migration (Figure S3C,D). cDC1s are critically involved in antigen cross‐presentation and the activation of CD8^+^ T cells against pathogens [13, 14]. Notably, CCL5 well facilitated the migration of CD11c^+^ CD103^+^ cDC1 cells (Figure 1F).

Next, we verified whether the reduction of CCL5 in hepatic NK cells impaired the migration of DCs. We found CCL5 deficiency decreased the ability of NK cells to promote BMDC migration in vitro (Figure 1G and Figure S4). Although CCL5 promoted the migration of DCs, cDC1 and cDC2 subsets in BMDCs derived from both WT and HBV‐carrier mice, the BMDCs of HBV‐carrier mice, especially cDC1 subset, presented impaired migration ability and responsiveness to CCL5 compared with BMDCs of WT mice (Figure 1H). Meanwhile, the expression of CCL5‐associated high‐affinity receptors, especially CCR1 and CCR5, were decreased in DCs, cDC1 and cDC2 subsets in BMDCs derived from HBV‐carrier mice (Figure S5).

In addition to NK cells, multiple immune cells such as T cells and B cells also produce CCL5 to recruit immune cells to the sites of inflammation [18, 26]. The mouse gene expression microarray data for different immune cells from the Immunological Genome Project (ImmGen, GSE15907) revealed that NK cells are the main source of CCL5, and CCL5 is a prominent chemokine secreted by NK cells (Figure 1I). Significantly, compared with other immune cells such as T cells and DCs, hepatic NK cells from WT mice expressed substantially higher levels of CCL5 (Figure 1J). Additionally, compared to hepatic NK cells from WT mice, NK cells from HBV‐carrier mice showed low production of CCL5 (Figure 1J). These findings suggest that chronic HBV infection disrupts the production of CCL5 mainly by hepatic NK cells and impairs the responsiveness of DCs to CCL5, limiting the intrahepatic accumulation of DCs especially that of cDC1s.

### 3.3 Deficiency of CCL5 in NK cells Dampens the Intrahepatic DC Accumulation and T‐Cell‐Mediated Anti‐HBV Activity in Vivo

**FIGURE 2:**
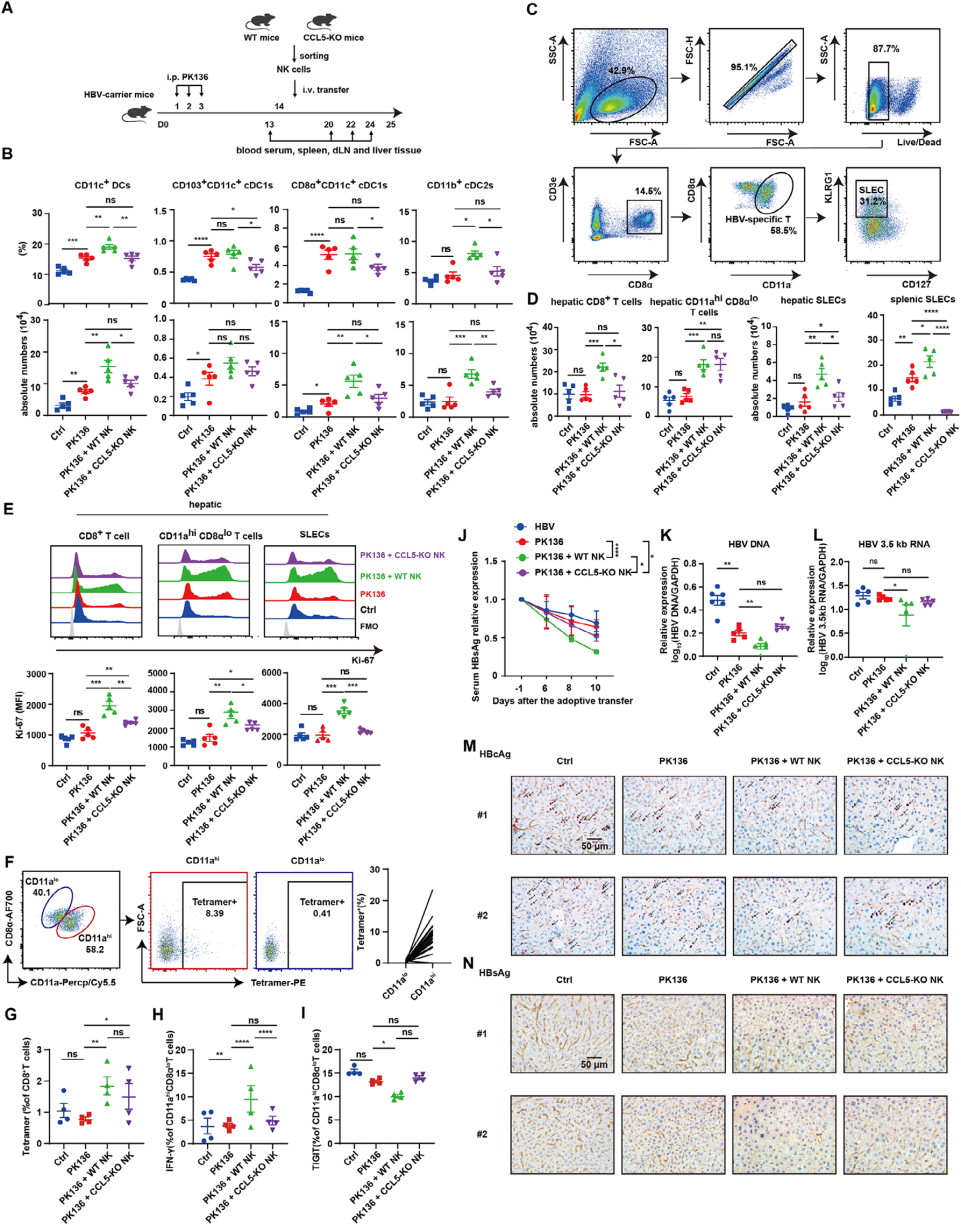
CCL5‐deficient NK cells impair the hepatic accumulation of DCs and anti‐HBV capacity of CD8^+^ T cells. HBV‐carrier mice were treated with anti‐PK136 to deplete NK cells and then intravenously administered 5 × 10^5^ WT or CCL5‐KO NK cells. These mice were sacrificed 10 days after transfer. (A) The adoptive transfer protocol for NK cells. (B) Flow cytometric analysis of the numbers of DCs, CD103^+^ cDC1s, CD8α^+^ cDC1s, and CD11b^+^ cDC2s in liver tissues. (C) Gating strategy for CD8^+^ T cells, HBV‐specific CD8^+^ T cells, and SLECs in the liver. (D) Absolute numbers of hepatic CD8^+^ T cells and HBV‐specific CD8^+^ T cells, hepatic and splenic SLECs. (E) Flow cytometric analysis of Ki‐67 expression in hepatic CD8^+^ T cells, HBV‐specific CD8^+^ T cells, and SLECs. (F) Gating strategy and flow cytometric analysis of HBsAg MHC‐I Tetramer positive cells in CD11a^hi^ CD8^+^ T cells (HBV‐specific CD8^+^ T cells) or CD11a^lo^ CD8^+^ T cells in liver tissues. (G) Flow cytometric analysis of the percentages of HBsAg MHC‐I Tetramer positive cells in CD8^+^ T cells. (H,I) Flow cytometric analysis of the percentages of IFN‐γ (H) and TIGIT (I) positive cells in HBV‐specific CD8^+^ T cells. (J) Serum was collected at the indicated time points, and the relative levels of serum HBsAg were determined by CLIA. (K‐L) RT‐qPCR analysis of intrahepatic HBV DNA (K) and the 3.5 kb RNA intermediate product of HBV (L). (M,N) HBcAg (M) and HBsAg (N) expression in hepatocytes was determined by immunohistochemical (IHC) staining (scale bar, 50 µm). The data are presented as the means ± SEMs (*n* ≥ 4). ns, not significant. ^*^ *p* < 0.05, ^**^ *p* < 0.01, ^***^ *p* < 0.001, ^****^ *p* < 0.0001.

To confirm the effects of NK cells on DC liver accumulation in vivo, hepatic NK cells were sorted from WT and CCL5‐KO mice, respectively, and then adoptively transferred into recipient HBV‐carrier mice that NK cells were pre‐depleted by anti‐PK136 (Figure 2A). Compared with mice NK cell‐depleted, the adoptive transfer of WT NK cells increased the number of hepatic total DCs and cDC subsets, whereas the adoptive transfer of CCL5‐KO NK cells did not significantly affect the intrahepatic accumulation of DCs (Figure 2B).

The stable magnitude and functionality of HBV‐specific CD8^+^ T cell responses play a crucial role in effector and memory responses against HBV. Interestingly, we found that the adoptive transfer of WT NK cells significantly increased the number of hepatic CD8^+^ T cells, HBV‐specific CD8^+^ T cells (CD11a^hi^ CD8α^lo^) [27] and SLECs (CD127^lo^ KLRG1^hi^) [9] in recipient HBV‐carrier mice (Figure 2C,D), accompanied by increased expression of Ki‐67 (Figure 2E), whereas the adoptive transfer of CCL5‐deficient NK cells had no significant effect on T cells. Subsequently, we used HBsAg (VWLSVIWM) peptide‐specific H2‐Kb tetramer to further characterize the specificity and functionality of HBsAg‐specific CD8^+^ T cells upon the adoptive transfer of NK cells. The results showed the frequency of tetramer^+^ CD11a^hi^ CD8α^lo^ cells was significantly higher than tetramer^+^ CD11a^lo^ CD8α^hi^ cells (Figure 2F), confirming the CD11a^hi^ CD8α^lo^ cells were HBV‐specific cells. And, we found that adoptive transfer of WT NK cells significantly increased the proportion of tetramer^+^ CD8^+^ T cells, accompanied with the increased IFN‐γ and decreased TIGIT expression by HBV‐specific CD8^+^ T cells compared with those in the NK cell‐depleted mice (Figure 2G–I). Although the transfer of CCL5‐deficient NK cells slightly increased the frequency of tetramer^+^ CD8^+^ T cells, they had no significant effects on the functionality of these antigen‐specific CD8^+^ T cells.

Importantly, the adoptive transfer of WT NK cells significantly reduced serum HBsAg (Figure 2J), intrahepatic HBV DNA (Figure 2K), HBV RNA (Figure 2L), HBcAg (Figure 2M), and HBsAg (Figure 2N) levels in the recipient HBV‐carrier mice, while the transfer of CCL5‐deficient NK cells exhibited weaker inhibitory effects on HBV than WT NK cells. Notably, the anti‐HBV effects induced by the transfer of WT NK cells were eliminated in the presence of anti‐CD8 antibody (Figure S6). These results suggest that CCL5‐deficient NK cells fail to mediate the chemotaxis and migration of DCs, impairing CD8^+^ T‐cell function and the ability to clear HBV.

### 3.4 Chronic HBV Infection Decreases the Levels of Hepatic cNK Cells, the Main Producers of CCL5

**FIGURE 3:**
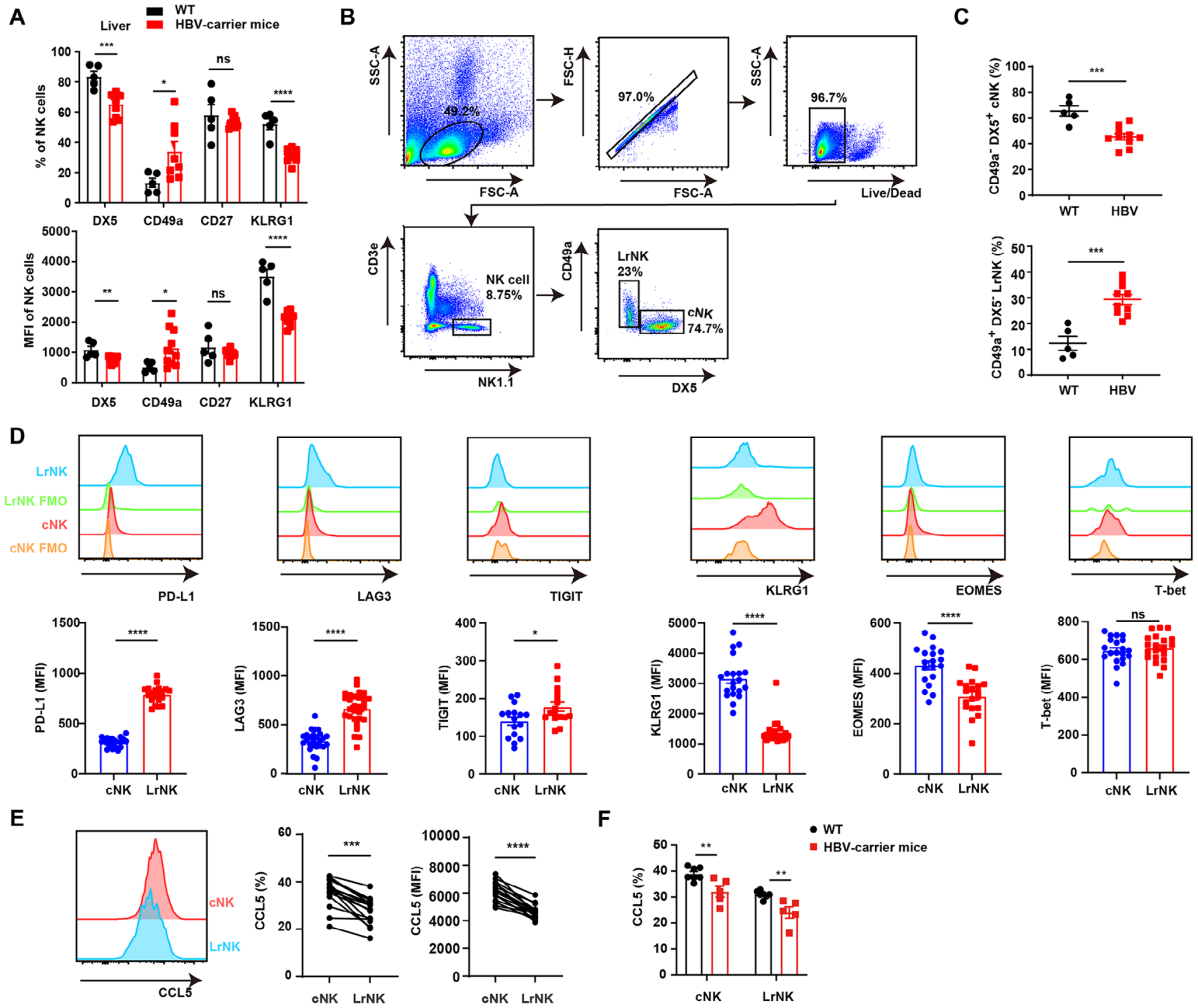
Chronic HBV infection reduces the number of hepatic cNK cells, the main source of CCL5. (A) The expression levels of DX5, CD49a, CD27, and KLRG1 on hepatic NK cells from WT and HBV‐carrier mice were analyzed via flow cytometry. (B) Gating strategy for hepatic NK cells, cNK cells, and LrNK cells. (C) The frequency of hepatic cNK and LrNK cells in WT and HBV‐carrier mice was analyzed by flow cytometry. (D) The expression levels of PD‐L1, LAG3, TIGIT, KLRG1, T‐bet, and EOMES in hepatic cNK and LrNK cells from WT mice were analyzed via flow cytometry. (E) The expression levels of CCL5 in hepatic cNK cells and LrNK cells from WT mice were analyzed via flow cytometry. (F) The expression levels of CCL5 in hepatic cNK cells and LrNK cells from WT and HBV‐carrier mice were analyzed via flow cytometry. The data are presented as the means ± SEMs (*n* ≥ 5). ns, not significant. ^*^ *p* < 0.05, ^**^ *p* < 0.01, ^***^ *p* < 0.001, ^****^ *p* < 0.0001.

Murine liver NK cells contain two distinct subpopulations: CD49a^−^ DX5^+^ conventional NK (cNK) cells and CD49a^+^ DX5^−^ liver‐resident NK (LrNK) cells, which have different functions and transcription factor profiles [28, 29]. Then, we tried to identify which subset of hepatic NK cells is the major source of CCL5. Compared with WT mice, HBV‐carrier mice presented downregulated DX5 expression and upregulated CD49a expression in NK cells (Figure 3A), and the proportion of cNK cells was significantly lower while the proportion of LrNK cells was significantly higher in the livers of HBV‐carrier mice than in WT mice (Figure 3B,C). Further flow cytometric analysis confirmed the expression of IFN‐γ, CD69, Ki‐67, and EOMES was significantly downregulated in both cNK and LrNK subsets, while T‐bet expression did not show significant change, which was consistent with the changes observed in total NK cells (Figure S2B–G). Additionally, chronic HBV infection induced the exhaustion and disturbed the differentiation of NK cells, characterized as the increase of PD‐L1 and LAG‐3, the decrease of KLRG1, and the cNK differentiation‐related transcription factor EOMES [17] in NK cells, cNK cells, and LrNK cells (Figure S7). Notably, compared with cNK cells, LrNK cells expressed high levels of PD‐L1, LAG3 and TIGIT, and low levels of KLRG1 and EOMES (Figure 3D). Although both cNK cells and LrNK cells produced CCL5, the levels of CCL5 in cNK cells were higher than in LrNK cells (Figure 3E). Chronic HBV infection inhibited CCL5 production in both cNK and LrNK cells (Figure 3F). These findings indicate that cNK cells is the major producers of CCL5 in the liver, but the number and CCL5‐secreting ability of cNKs are inhibited in chronic HBV infection.

### 3.5 Hepatic cNK Cells Promote the Accumulation and Activation of DCs

**FIGURE 4:**
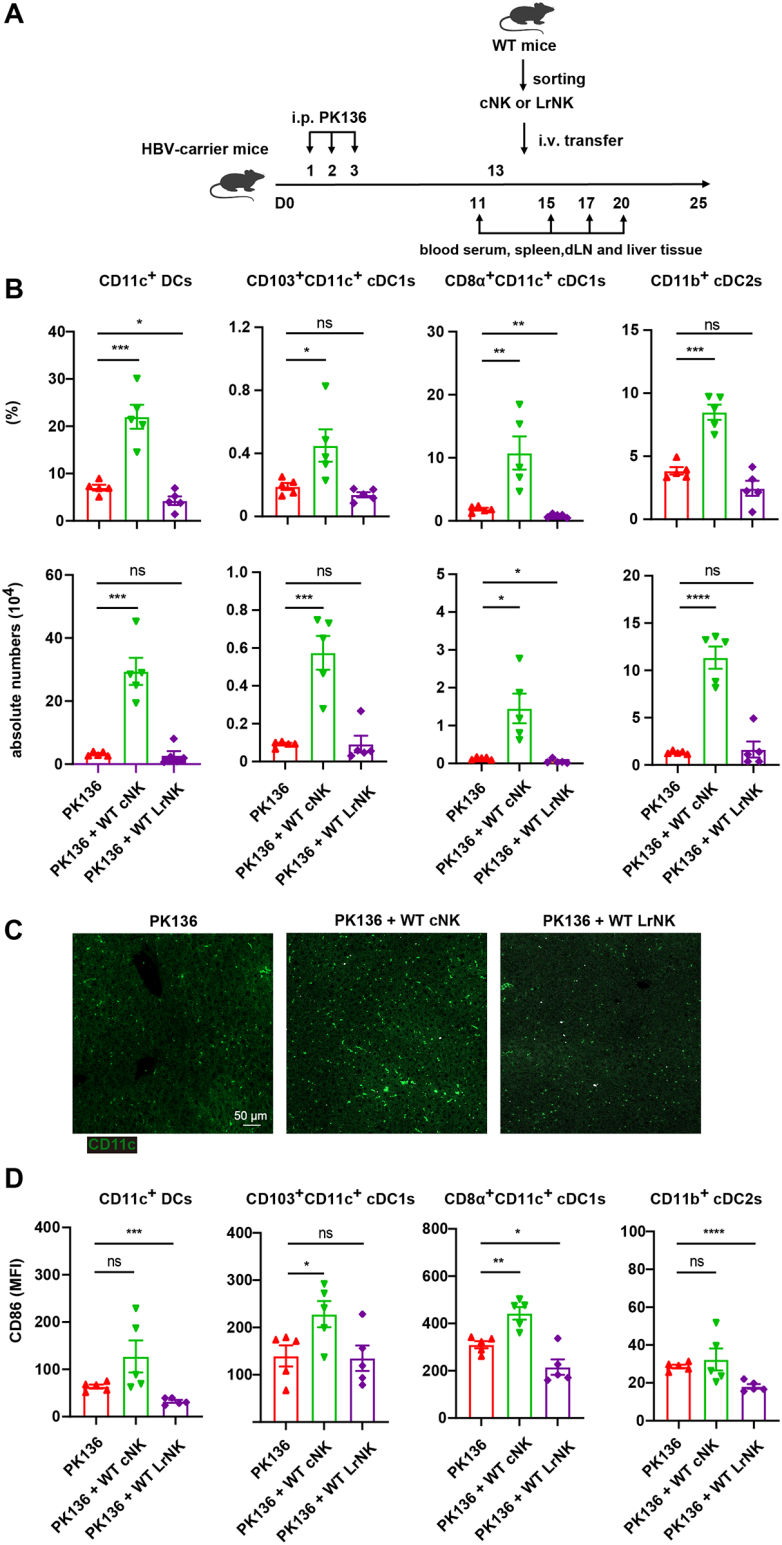
Comparison of cNK and LrNK cells in driving the intrahepatic accumulation and activation of DCs. The cNK and LrNK cells were sorted from the livers of WT mice by Moflo Astrios EQ. HBV‐carrier mice were treated with anti‐PK136 to deplete NK cells and then intravenously administered 5 × 10^4^ cNK and LrNK cells, respectively. These mice were sacrificed 12 days after transfer. (A) The adoptive transfer protocol for NK cells. (B) The frequency and absolute numbers of DCs, CD103^+^ cDC1s, CD8α^+^ cDC1s, and CD11b^+^ cDC2s were determined via flow cytometry. (C) Immunofluorescence analysis of hepatic DCs (green). (D) Flow cytometric analysis of CD86 expression on hepatic DCs, CD103^+^ cDC1s, CD8α^+^ cDC1s, and CD11b^+^ cDC2s. The data are presented as the means ± SEMs (*n* ≥ 4). ns, not significant. ^*^ *p* < 0.05, ^**^ *p* < 0.01, ^***^ *p* < 0.001, ^****^ *p* < 0.0001.

To further confirm the role of cNK and LrNK cells in regulating DCs, cNK cells and LrNK cells were isolated from the livers of WT mice, and adoptively transferred into recipient HBV‐carrier mice, respectively (Figure 4A). Compared with NK cell‐depleted mice, the proportions and numbers of hepatic total DCs, cDC1 and cDC2 subsets were greater in the mice adoptively transferred with cNK cells (Figure 4B,C), accompanied with the increased expression of CD86 (Figure 4D). In contrast, the adoptive transfer of LrNK cells inhibited hepatic accumulation and activation of DCs (Figure 4A–D). These data suggest that cNK cells promote the accumulation and maturation of DCs in the liver, whereas LrNK cells play opposite roles.

### 3.6 cNK Cells Facilitate the Differentiation and Function of CD8^+^ T Cells

**FIGURE 5:**
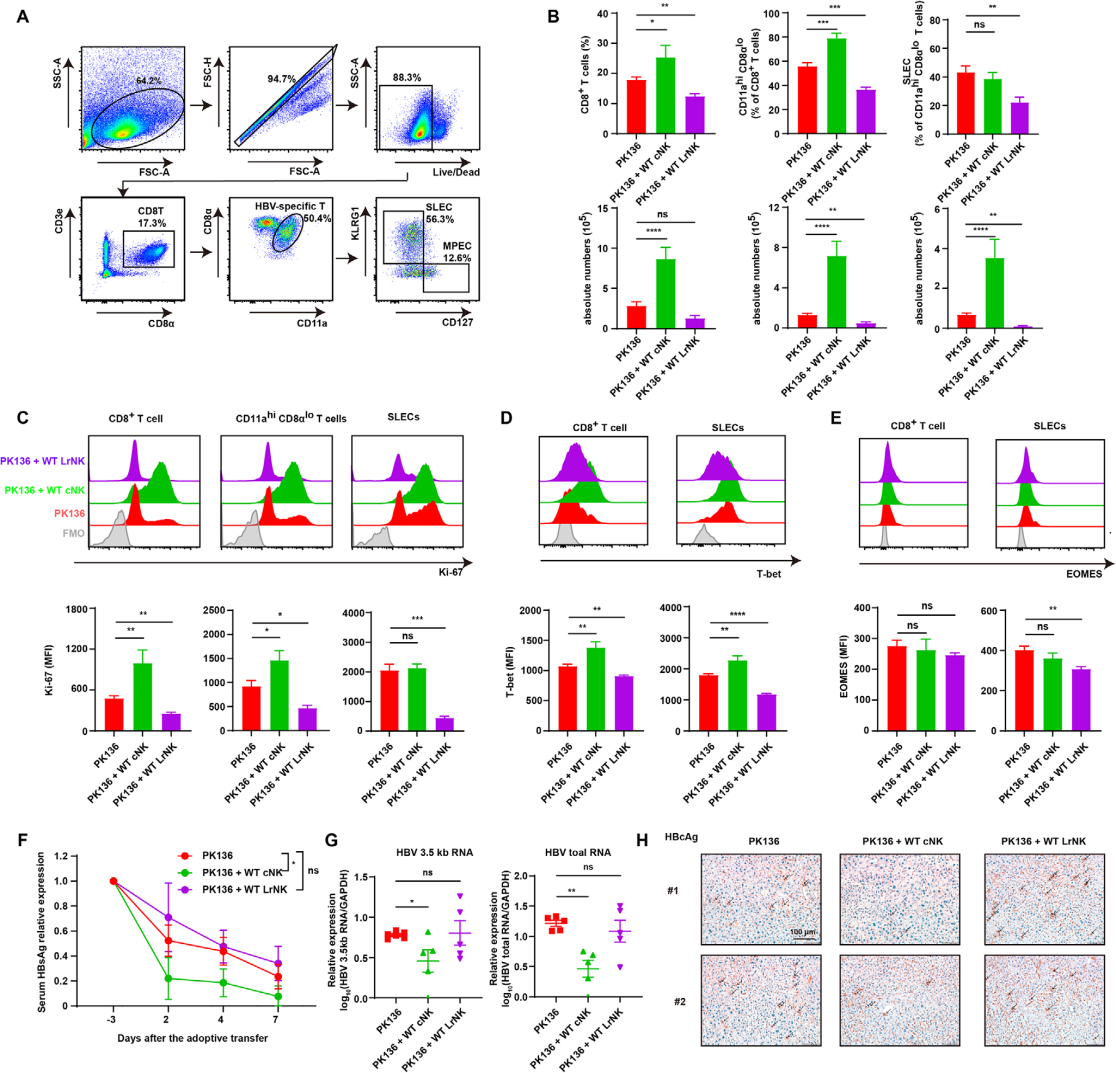
cNK cells promote the proliferation and differentiation of CD8^+^ T cells and the clearance of HBV. The cNK and LrNK cells were sorted from the livers of WT mice by Moflo Astrios EQ. HBV‐carrier mice were treated with anti‐PK136 to deplete NK cells and then intravenously transferred with 5 × 10^4^ cNK and LrNK cells, respectively. These mice were sacrificed 12 days after transfer. (A) Gating strategy for hepatic CD8^+^ T cells, HBV‐specific CD8^+^ T cells, and SLECs. (B) Flow cytometric analysis of the frequencies and numbers of hepatic CD8^+^ T cells, HBV‐specific CD8^+^ T cells, and SLECs. (C) Flow cytometric analysis of Ki‐67 expression in hepatic CD8^+^ T cells, HBV‐specific CD8^+^ T cells, and SLECs. (D‐E) Flow cytometric analysis of the levels of T‐bet (D) and EOMES (E) in hepatic CD8^+^ T cells and SLECs. (F) Serum was collected at the indicated time points, and the relative levels of serum HBsAg were determined by CLIAs. (G) RT‐qPCR analysis of the 3.5 kb intrahepatic HBV intermediate RNA product and total HBV RNA. (H) HBcAg expression in hepatocytes was detected via IHC staining (scale bar, 100 µm). The data are presented as the means ± SEMs (*n* ≥ 4). ns, not significant. ^*^ *p* < 0.05, ^**^ *p* < 0.01, ^***^ *p* < 0.001, ^****^ *p* < 0.0001.

We subsequently analyzed the effects of cNK and LrNK cells on hepatic HBV‐specific T cells in HBV‐carrier mice. The results showed that the adoptive transfer of cNK cells significantly increased the total number of hepatic CD8^+^ T cells, HBV‐specific CD8^+^ T cells, and SLECs, accompanied by increased expression of Ki‐67; however, the adaptive transfer of LrNK cells exhibited suppressive effects on the accumulation and Ki‐67 expression of T cells in the liver (Figure 5A–C). Meanwhile, the adoptive transfer of cNK cells also significantly increased HBV‐specific CD8^+^ T cells in spleen (Figure S8). T‐bet and EOMES are key regulators that control the differentiation of effector T cells and memory T cells [30]. We found the adoptive transfer of cNK cells upregulated T‐bet in hepatic CD8^+^ T cells and SLECs, whereas LrNK cells significantly suppressed T‐bet expression (Figure 5D); but, neither cNK cells nor LrNK cells significantly affected EOMES levels in CD8^+^ T cells or SLECs (Figure 5E). Notably, the transfer of cNK cells augmented anti‐HBV responses, as indicated by the obvious reduce of serum HBsAg (Figure 5F), intrahepatic HBV RNA (Figure 5G), and HBcAg (Figure 5H) levels in the recipient HBV‐carrier mice. However, LrNK cells inhibited HBV‐specific T‐cell responses, suppressing HBV clearance (Figure 5F–H). These results indicate that hepatic cNK cells promote the proliferation and differentiation of HBV‐specific CD8^+^ T cells, enhancing HBV clearance.

### 3.7 Chronic HBV Infection Inhibits CCL5 Secretion by NK Cells Through Adenosine Signaling in HBV‐Carrier Mice

Adenosine, through the activation of cyclic adenosine monophosphate (cAMP), suppresses inflammatory responses to protect against immune‐mediated damage, but a persistent increase in adenosine supports the formation of an immunosuppressed niche [31, 32]. Next, we investigated whether chronic HBV infection inhibited CCL5 secretion by NK cells through the adenosine signaling pathway. Adenosine is produced via stepwise hydrolysis of extracellular adenosine triphosphate (ATP) by the ectonucleotidases CD39 (Entpd1) and CD73 (Nt5e). We found that the levels of both CD39 and CD73 were significantly higher in the hepatic MNCs and NK cells of HBV‐carrier mice than in those of WT mice (Figure S9A–E). These results indicate that adenosine signaling may be involved in regulating NK cell functions during chronic HBV infection.

**FIGURE 6:**
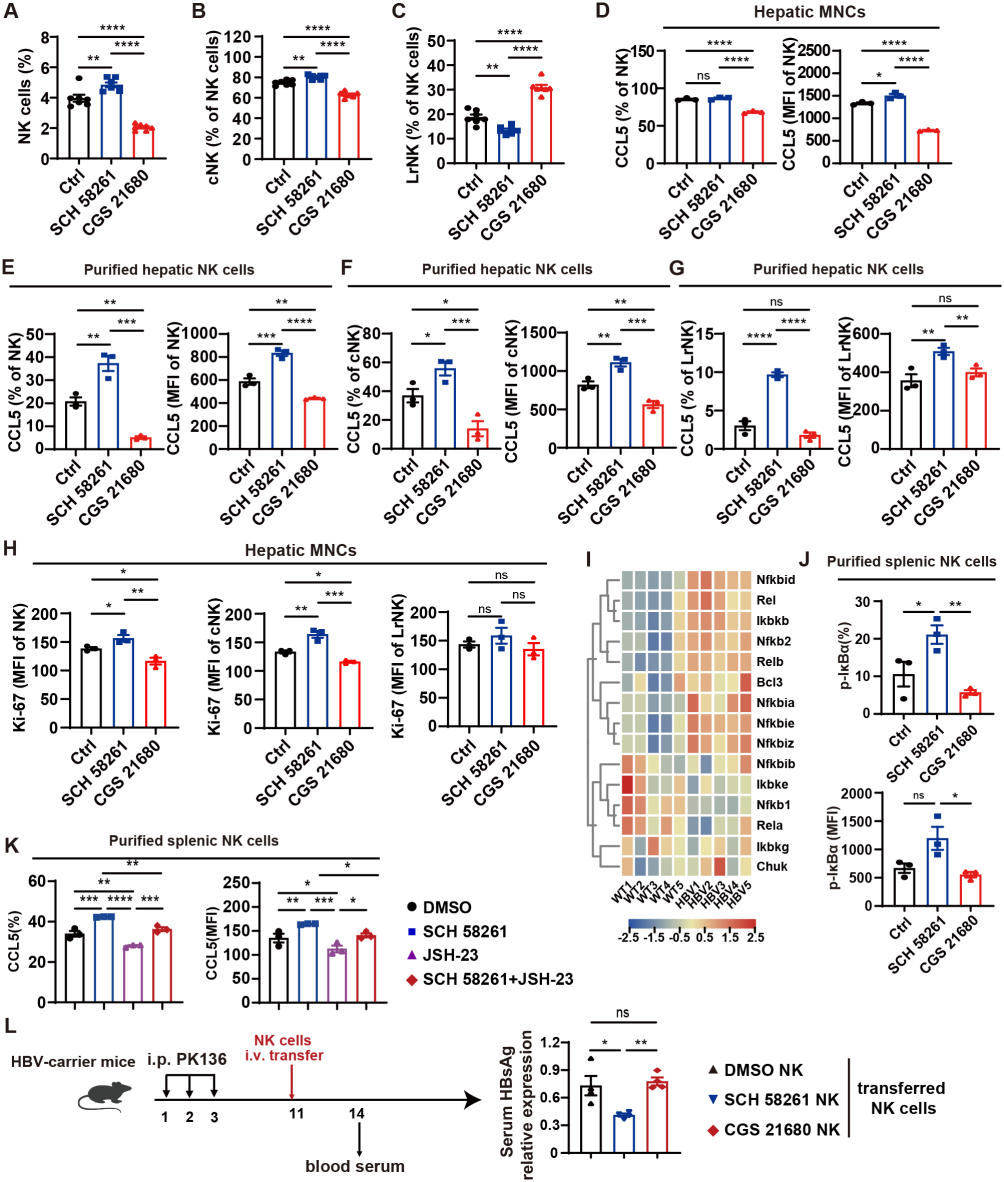
Chronic HBV infection inhibited CCL5 production by NK cells through adenosine signaling in HBV‐carrier mice. (A–C) HBV‐carrier mice were intraperitoneally injected with DMSO, ADORA2A antagonist SCH 58261 (1 mg/kg), or ADORA2A agonist CGS 21680 (1 mg/kg) once every three days. These mice were sacrificed 24 h after the final injection, and the percentages of hepatic NK cells (A), cNK cells (B), and LrNK cells (C) were determined via flow cytometry. (D) Hepatic MNCs from WT mice were treated with DMSO, SCH 58261 (1 µm), or CGS 21680 (1 µm) for 24 h, then the expression of CCL5 in NK cells was determined by flow cytometry. (E–G) Hepatic NK cells sorted from HBV‐carrier mice via the MojoSort Mouse NK Cell Isolation Kit were treated with DMSO, SCH 58261 (1 µm), or CGS 21680 (1 µm) for 24 h, then the CCL5 level in the NK cells (E), cNK cells (F), and LrNK cells (G)was determined via flow cytometry. (H) Hepatic MNCs from WT mice were treated with DMSO, SCH 58261 (1 µm) or CGS 21680 (1 µm) for 24 h, then the expression of Ki‐67 in NK cells, cNK cells and LrNK cells was determined by flow cytometry. (I) Landscape heatmap showing the profile of genes related to NFκB signaling in hepatic NK cells from WT and HBV‐carrier mice via RNA‐seq. (J) Splenic NK cells from HBV‐carrier mice were treated with DMSO, SCH 58261 (1 µm) or CGS 21680 (1 µm) for 12 h, and the level of *p*‐IκBα in splenic NK cells was determined by flow cytometry. (K) Splenic NK cells from HBV‐carrier mice were treated with DMSO, SCH 58261 (1 µm), JSH‐23 (10 µm), or SCH 58261 (1 µm) plus JSH‐23 (10 µm) for 24 h, then the level of CCL5 in NK cells was determined by flow cytometry. (L) HBV‐carrier mice were treated with anti‐PK136 to deplete NK cells and then intravenously administered 1 × 10^5^ NK cells pretreated with DMSO, SCH 58261 (1 µm) or CGS 21680 (1 µm). These mice were sacrificed 14 days after transfer, and the relative levels of serum HBsAg were determined via CLIAs. The data are presented as the means ± SEMs (*n* ≥ 3). ns, not significant. ^*^ *p* < 0.05, ^**^ *p* < 0.01, ^***^ *p* < 0.001, ^****^ *p* < 0.0001.

There are four subtypes of adenosine receptors, ADORA1, ADORA2A, ADORA2B, and ADORA3, these receptors are differentially expressed in immune cells and have different functions [32, 33]. RNA‐seq analysis revealed that the ADORA2A level was significantly greater than that of other receptor subtypes in both hepatic NK cells from WT mice and HBV‐carrier mice (Figure S9F). To confirm whether ADORA2A‐medited signaling influence NK subsets, HBV‐carrier mice were treated with ADORA2A antagonist and ADORA2A agonist, respectively. We observed the percentages of hepatic NK cells and cNK cells were significantly increased upon ADORA2A antagonist treatment, whereas the percentage of LrNK cells was decreased (Figure 6A–C). However, ADORA2A agonist displayed the opposite effects to ADORA2A antagonist.

Subsequently, we confirmed the direct regulatory roles of ADORA2A signaling on NK cells in vitro. Subsequently, we confirmed the direct regulatory role of ADORA2A signaling in NK cells in vitro. ADORA2A antagonist significantly upregulated CCL5 expression in total hepatic NK cells, cNK cells, and LrNK cells from HBV‐carrier mice, whereas ADORA2A agonist exerted the opposite effect in total hepatic NK cells and cNK cells, but not in LrNK cells (Figure 6D–G). Consistent results were obtained in NK cells from splenic mononuclear cells (MNCs) and purified splenic NK cells treated with the ADORA2A antagonist or agonist (Figure S9G,H). In parallel, Ki‐67 expression in total hepatic NK cells and the cNK subset from HBV‐carrier mice was significantly increased by ADORA2A antagonist and reduced by ADORA2A agonist, whereas the LrNK subset remained largely unaffected (Figure 6H). Similar effects were observed in splenic NK cells (Figure S9I). These findings suggest that ADORA2A‐mediated adenosine signaling suppresses NK‐cell proliferation during chronic HBV infection, predominantly by impairing the proliferative capacity of the cNK cells.

Adenosine has been shown to inhibit NFκB activation and the proinflammatory response [32, 34]. RNA‐seq analysis revealed that, compared with WT NK cells, NK cells from HBV‐carrier mice presented decreased expression of p50 (Nfκb1) and p65 (RELA), but significantly increased expression of IκBα (Nfkbia, a major suppressor of NFκB) (Figure 6I). Interestingly, the ADORA2A antagonist significantly increased the phosphorylation level of IκBα, whereas ADORA2A agonist downregulated it in NK cells from HBV‐carrier mice (Figure 6J). Furthermore, ADORA2A antagonist promoted the production of CCL5 by NK cells from HBV‐carrier mice, but the NFκB inhibitor JSH‐23 resisted the effect of ADORA2A antagonist on CCL5 (Figure 6K). Ultimately, NK cells pretreated with ADORA2A antagonist or agonist were adoptively transferred into recipient HBV‐carrier mice, respectively. Compared with NK cell‐depleted mice, we found that the transfer of ADORA2A antagonist‐treated NK cells could significantly reduce serum HBsAg levels, while the transfer of ADORA2A agonist‐treated NK cells did not show significant influence on serum HBsAg levels (Figure 6L). These results suggest that chronic HBV infection inhibits CCL5 production in NK cells through adenosine signaling.

### 3.8 The Production of CCL5 by cNK Cells is Suppressed in Patients With CHB via the ADORA2A/NFκB Pathway

**FIGURE 7:**
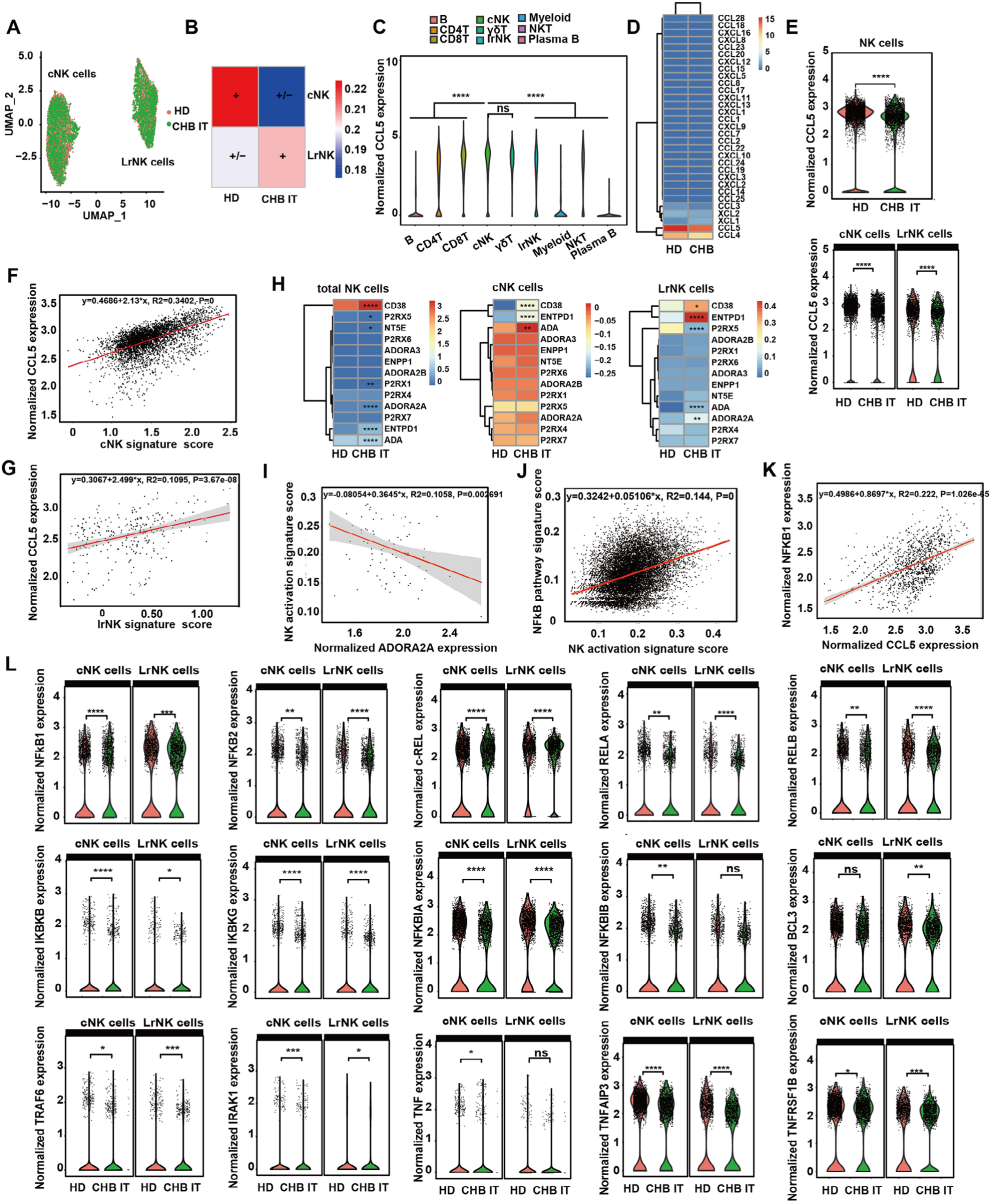
Chronic HBV infection inhibits CCL5 production by NK cells through adenosine signaling in immune‐tolerant CHB patients. (A) UMAP plots of hepatic cNK and LrNK cells from HDs and immune‐tolerant CHB patients obtained from the GEO database (GSE182159) are shown. (B) Preference of cNK and LrNK cell clusters among HDs and immune‐tolerant CHB patients was estimated via the STARTRAC‐dist index. +, 0.2 < Ro/e < 0.22; +/−, 0.18 < Ro/e < 0.2; Ro/e denotes the ratio of observed to expected cell numbers. (C) CCL5 expression in the indicated hepatic immune cells. (D) Landscape heatmap showing the patterns of chemokines in hepatic NK cells between HDs and immune‐tolerant CHB patients via scRNA‐seq. (E) CCL5 expression in total NK cells (upper) and cNK and LrNK cells (lower) from HDs and immune‐tolerant CHB patients was quantified. (F) Correlation between CCL5 expression and the cNK signature score. The cNK signature genes used for this analysis included FCGR3A, GNLY, and GZMB. (G) Correlation between CCL5 expression and the LrNK signature score. The LrNK signature genes used for this analysis included NCAM1, EOMES, ITGA1, and CXCR6. (H) Landscape heatmaps showing the expression of adenosine pathway members in hepatic total NK cells, cNK cells, and LrNK cells from HDs and immune‐tolerant CHB patients. Color indicates the per‐group mean expression (mean normalized expression for the total NK cell panel; mean z‐scored expression for the cNK and LrNK cell panels). Asterisks indicate genes with significantly different expression between HDs and immune‐tolerant CHB patients, as determined by the Wilcoxon rank‐sum test. (I) Correlation of ADORA2A expression and the NK cell activation signature among NK cells. The NK cell activation signature genes used for this analysis included BCL2, CCL3, CCL4, CCR5, CD69, CXCL10, FOXK1, GBP4, GZMB, ICAM1, IFNG, IL12RB1, IL12RB2, IL2RA, IRF1, IRF7, IRF8, KLF11, KLF13, KLRG1, NFIL3, NOTCH1, NR4A1, NR4A2, NR4A3, SOCS1, SOCS3, STAT1, STAT2, STAT4, and TBX21. (J) Correlations between NFκB1 expression and the NK cell activation signature among NK cells. (K) Correlation of NFκB1 and CCL5 expression among NK cells. (L) The expression of NFκB members and target gene transcripts, such as NFκB1, NFκB2, c‐REL, RELA, RELB, TNFAIP3, and TNFRSF1B, in hepatic cNK cells from HDs and immune‐tolerant CHB patients. ns, not significant. ^*^ *p* < 0.05, ^**^ *p* < 0.01, ^***^ *p* < 0.001, ^****^ *p* < 0.0001.

To determine whether hepatic NK cells in patients with CHB exhibited phenotypes similar to those observed in HBV‐carrier mice, we analyzed the transcriptional changes and heterogeneity of hepatic NK cells in HDs and immune‐tolerant CHB patients via a published scRNAseq dataset (GSE182159) [25]. First, we identified two main NK cell subsets: cNK subcluster (CD16^+^ CD56^dim^) and LrNK subcluster (CD49a^+^ CD16^−^), and the cNK subcluster presented higher expression of CCL5 than LrNK subcluster (Figure S10A,B) [20, 35]. Consistent with the findings in HBV‐carrier mice, the proportion of hepatic cNK subcluster was lower and the LrNK subcluster was higher in immune‐tolerant CHB patients than in HDs (Figure 7A,B). The CCL5 mRNA of intrahepatic CD45^+^ immune cells significantly decreased in immune‐tolerant CHB patients compared with HDs (Figure S11). Further analysis showed CCL5 expressed in hepatic cNK cells was higher than other immune cells such as LrNK cells, T cells, and B cells (Figure 7C). Notably, the chemokine profile of hepatic NK cells was altered by chronic HBV infection (Figure 7D), and CCL5 levels in hepatic NK cells, cNK subcluster, and LrNK subcluster of immune‐tolerant CHB patients were obviously lower than in HDs (Figure 7E). Additionally, we detected a positive correlation between the cNK signature and CCL5 expression (Figure 7F, R^2^ = 0.3402) but a weaker correlation between the LrNK signature and CCL5 expression (Figure 7G, R^2^ = 0.1095), indicating that cNK cells are the main source of CCL5 in human liver.

Next, we investigated whether adenosine signaling is involved in NK cell activation and CCL5 expression in immune‐tolerant CHB patients. Compared with healthy donors, CD38, ENTPD1 (CD39), NT5E (CD73), ADA, and ADORA2A were significantly upregulated in total NK cells from CHB IT patients. In both cNK and LrNK cells, CD38, ENTPD1, ADA, and NT5E were significantly upregulated, whereas ADORA2A was significantly upregulated only in LrNK cells (Figure 7H). Moreover, the expression level of ADORA2A negatively correlated with NK cell activation (Figure 7I), while NFκB activation was positively correlated with the NK cell activation signature (Figure 7J) and CCL5 gene expression (Figure 7K). However, the transcripts of NFκB family members and target genes, including NFκB1, NFκB2, c‐REL, RELA, RELB, TNF Alpha‐Induced Protein 3 (TNFAIP3), and TNF Receptor Superfamily Member 1B (TNFRSF1B), were all reduced in cNK cells from CHB patients (Figure 7L). These data confirm that chronic HBV infection suppresses the production of CCL5 by human NK cells via ADORA2A/NFκB signaling pathway.

To gain insight into the regulatory programs of NK cells in regulating the accumulation and function of hepatic DCs in immune‐tolerant CHB patients, we utilized CellPhoneDB to probe the potential cell‒cell interactions between NK cells and other CD45^+^ immune cells, especially DCs. Significantly, the interaction between NK cells and DCs was predicted to be mediated via the CCL5‐CCR1 receptor (Figure S12), indicating that the production of CCL5 by cNK cells is suppressed in patients with CHB through the adenosine signaling pathway.

## 4 Discussion

Chronic HBV infection leads to the dysfunction of DCs and HBV‐specific CD8^+^ T cells, resulting in persistent HBV infection [6, 7]. Previous reports have also shown that the proportion of DCs is negatively correlated with HBsAg levels [36]. Moreover, the HBV‐carrier mice presented decreased DC1 and cDC2 subsets, accompanied by the downregulation of MHC‐I, MHC‐II, CD80 and CD86, indicating that chronic HBV infection affects the differentiation and function of HBV‐specific CD8^+^ T cells by impairing the functions of DCs.

The immune microenvironment of the liver is a complex network of immune cells. In addition to direct cytotoxicity to HBV‐infected hepatocytes, NK cells can also interact with other immune cells, such as DCs and CD8^+^ T cells, where they perform modulatory functions. Studies have shown that NK cells produce chemokines such as XCL1, CCL5, and FLT3L to recruit and promote the differentiation of cDC1s that highly express the corresponding receptors XCR1, CCR1, and CCR5 in the tumor microenvironment, promoting antitumor immunity [18, 19, 20, 37]. Studies have confirmed that the presence of cDC1s and NK cells in tumors is positively associated with CD8^+^ T‐cell infiltration, survival, and responsiveness to anti‐PD1 treatment in patients with cancer [18, 19, 20]. In this study, we observed the decreased number and dysfunction of DCs in HBV‐carrier mice, accompanied by impaired NK cell activation and maturation. Therefore, we speculated that chronic HBV infection‐induced abnormalities in NK cells contributed to the impaired hepatic accumulation and function of DCs. RNA‐seq and scRNA‐seq analyses revealed that chronic HBV infection disrupted the chemokine profile of NK cells. CCL5 was the most highly expressed gene, and its expression was strongly decreased by chronic HBV infection in both mice and CHB patients. We demonstrated that CCL5 promotes the hepatic accumulation of DCs, predominantly the cDC1 subset, while CCL5‐deficient NK cells lose this ability both in vitro and in vivo, which in turn altered the terminal differentiation of HBV‐specific CD8^+^ T cells and HBV clearance. Therefore, NK cells play an important role in recruiting hepatic DCs and subsequent anti‐HBV effects, and the selective recruitment of cDC1s via CCL5 produced by NK cells is a key step in initiating adaptive antiviral immunity. Nevertheless, because anti‐NK1.1‐mediated depletion may also affect NKT cells, we further examined CCL5 expression in hepatic NKT cells during chronic HBV infection. We found that CCL5 expression in hepatic NKT cells was much lower than that in NK cells and was not significantly altered in HBV‐carrier mice (data not shown), suggesting a limited contribution of NKT cell‐derived CCL5 in our model. Future studies using more specific NK‐cell depletion models, such as NKp46‐Cre‐based systems, will be needed to further distinguish the contributions of NK cells and NKT cells to HBV replication control and DC recruitment during chronic HBV infection.

SLECs show stronger anti‐HBV effects than MPECs upon HBV infection [8, 9]. Importantly, we found that CCL5 deficiency in NK cells impaired the formation of hepatic SLECs and the ability to eliminate HBV in HBV‐carrier mice. A study revealed that downregulation of CCL5 receptors diminishes the responsiveness of cDC1s to CCL5, dampening the chemotaxis of cDC1s to tumors [18]. The CellPhoneDB assay revealed that the crosstalk between NK cells and DCs might be mediated via the CCL5‐CCR1 interaction in CHB patients. However, we found that chronic HBV infection suppresses the expression of CCL5‐associated high‐affinity receptors, especially CCR1, on DCs, which further exacerbates the impairment of intrahepatic accumulation of DCs caused by CCL5 deficiency in NK cells.

Hepatic NK cells can be divided into cNK cells and LrNK cells, which exhibit distinct gene expression profiles and functions in both mice and humans [29, 38, 39]. cNK cells express higher levels of cytotoxic molecules, whereas LrNK cells preferentially express inhibitory immune regulators, such as NKG2A, LAG3, and PD‐L1 [17]. Previous studies have shown that cNK cell‐derived IFN‐γ promotes CD8^+^ T cell‐mediated antiviral defense [16], whereas LrNK cells suppress T‐cell responses in a PD‐L1‐dependent manner in LCMV infection and ChAd‐HBV‐immunized mouse models [17, 40]. Consistently, we observed that intrahepatic LrNK cells from HBV‐carrier mice suppressed CD8^+^ T cells through the PD‐L1/PD‐1 axis, as evidenced by reduced IFN‐γ, TNF‐α, and Ki‐67 expression (Figure S13). In the present study, we further demonstrated that cNK cells expressed higher levels of CCL5 than LrNK cells, and the adoptive transfer of cNK cells promoted hepatic DC accumulation and maturation and facilitated HBV clearance. Meanwhile, the adoptive transfer of cNK cells significantly increased splenic HBV‐specific CD8^+^ T cells, which possibly was due to the peripheral redistribution of expanded intrahepatic CD8^+^ T cells. In contrast, the adoptive transfer of LrNK cells exerted opposite effects to those of cNK cells. However, chronic HBV infection decreased the number of hepatic cNK cells and increased the number of LrNK cells. Previous studies have shown that IL‐10 suppresses DC maturation and impairs antigen presentation and T‐cell priming [41]. Thus, our findings suggest that expansion of LrNK cells with low CCL5 expression disrupts hepatic DC accumulation and function, thereby impairing the terminal differentiation of HBV‐specific CD8^+^ T cells and contributing to persistent HBV infection. This effect may be associated with the elevated expression of inhibitory immune regulators in LrNK cells during chronic HBV infection, including NKG2A, LAG3, IL‐10, and PD‐L1.

In addition to contributing to immune evasion in the tumor microenvironment, adenosine has specific roles in driving the dysfunction of immune cells, such as T cells, macrophages, and NK cells, thereby affecting the progression of viral hepatitis [31, 42]. CD39 and CD73 metabolize extracellular ATP to adenosine, which mediates immunosuppression primarily by triggering ADORA2A receptors, resulting in the activation of cAMP‐responsive element‐binding protein 1 (CREB1) and subsequent inhibition of the NFκB pathway [32]. A recent study revealed that increased transcriptional activity of CREM promoted the dysfunction of HBV‐specific CD8^+^ T cells, contributing to persistent HBV infection [43]. Additionally, increased CD73 expression on NK cells can stimulate adenosine production and reduce the toxicity of NK cells, resulting in the progression of HCC [42, 44, 45]. In our study, chronic HBV infection increased the levels of CD39, CD73, and ADORA2A on hepatic NK cells, especially cNK cells, and the ADORA2A agonist suppressed NFκB activation that was positively correlated with NK cell activation signature and CCL5 production. Many studies have confirmed NF‐κB as a direct transcriptional regulator of CCL5 through promoter mutagenesis, reporter assays, and ChIP‐based analyses [46, 47, 48]. In our present study, we found ADORA2A signaling modulated IκBα phosphorylation and NF‐κB activation, while the pharmacological inhibition of NF‐κB obviously abolished the regulatory effect of ADORA2A signaling on CCL5 expression, supporting NF‐κB as a critical downstream mediator of ADORA2A that regulates CCL5 expression in NK cells. Compared with LrNK cells, cNK cells are the major producers of CCL5 and presented greater enrichment of NFκB family members and target gene transcripts, further confirming the role of the NFκB pathway in regulating CCL5 production by cNK cells. Importantly, antagonizing ADORA2A on NK cells increased the percentage of hepatic NK cells, especially cNK cells, and promoted CCL5 secretion and HBV clearance in HBV‐carrier mice. PBMCs from immune‐tolerant CHB patients presented decreased CCL5 production by NK cells (data not shown). Therefore, we believe that chronic HBV infection might impair the NK cell‐mediated chemotaxis of DCs via the ADORA2A/NFκB/CCL5 axis.

It should be noted that this study mainly focuses on the role of NK cell‐derived CCL5 in regulating cDC1 recruitment and antiviral immunity, and does not comprehensively address the complexity of the hepatic microenvironment. In addition to NK cells, multiple cell types including macrophages, T cells and hepatic stellate cells can also secrete CCL5; moreover, other chemokines, including CCL2, CCL3, CCL9, and CXCL12 [49, 50, 51], as well as immune cell populations such as neutrophils and monocytes [52, 53, 54], may participate in regulating DCs recruitment and function. Future studies will be required to define the interactions among these pathways and their collective impact on the NK‐DC‐T‐cell axis during chronic HBV infection. Notably, our transcriptomic analysis also revealed increased CXCL12 expression in hepatic NK cells from HBV‐carrier mice. Although CXCL12 expression was markedly lower than CCL5 expression in hepatic NK cells, its established roles in immune‐cell retention, fibrosis, and immunosuppressive niche formation [50, 55, 56] suggest that this chemokine may represent an additional mechanism contributing to the dysregulated hepatic immune microenvironment during chronic HBV infection. Whether CXCL12 functionally interacts with the ADORA2A/NF‐κB/CCL5 pathway identified in this study or modulates the NK–DC–T‐cell axis warrants further investigation. Although the expression level of CXCL12 was markedly lower than CCL5 in hepatic NK cells, given the established roles of CXCL12 in immune‐cell retention, fibrosis, and immunosuppressive niche formation, we speculate this chemokine may represent an additional mechanism contributing to the dysregulated hepatic immune microenvironment during chronic HBV infection. Whether CXCL12 functionally interacts with the ADORA2A/NF‐κB/CCL5 pathway identified in this study or influences the NK‐DC‐T‐cell axis warrants further investigation.

In conclusion, our study identifies that hepatic NK cells act as pivotal regulators involved in the differentiation and function of CD8^+^ T cells by governing the hepatic accumulation and maturation of cDC1s in chronic HBV infection. Actually, chronic HBV infection disrupts the balance between cNK cells and LrNK cells in the liver and cNK cells constitute the main cell type that produces CCL5, a process that is controlled by NFκB. NK cell‐derived CCL5 augmented the accumulation and activation of hepatic DCs, especially the cDC1s. However, chronic HBV infection triggers the ADORA2A/NFκB signaling pathway in NK cells to inhibit the activation of NFκB, reducing the number of hepatic cNK cells and suppressing CCL5 production, resulting in decreased hepatic accumulation and activation of DCs. Disruption of the interaction between the cNK and cDC1 subsets led to impaired function and terminal differentiation of HBV‐specific CD8^+^ T cells and persistent HBV infection. Strategies that target the ADORA2A/NFκB/CCL5 axis in cNK cells might be potential new treatments for chronic HBV infection. Since only AAV‐transduced‐HBV‐carrier mice were used in this study, the ADORA2A/NFκB pathway in hepatic NK cells of chronic HBV infection should be further evaluated in other models, such as HBV‐transgenic mice and human‐liver‐chimeric mice supporting HBV infection.

## Author Contributions

J.Z., Z.G.T., and H.J.Z. conceived and designed the study; H.J.Z., A.L.Y., Y.C.W., Y.T.Y., J.W., C.P.B., and Z.X.W. performed the experiments; H.J.Z., A.L.Y., Y.C.W., and Q.J.H. analyzed the data; J.Z. and H.J.Z. wrote the manuscript; and all the authors critically read and approved the final manuscript.

## Funding

This work was supported by the National Science Foundation of China (No.82471763, 82472832, and 82001687), Shandong Provincial Natural Science Foundation for The Excellent Youth Scholars (No. ZR2023YQ066, No. ZR2022YQ75), the Taishan Youth Scholar Fund of Shandong Province (tsqn202312058), Shandong Provincial Natural Science Foundation (No. ZR2024MH006), National Postdoctoral Program for Innovative Talents (No. BX20190192), and the Scientific Research Foundation of State Key Laboratory of Vaccines for Infectious Diseases, Xiang An Biomedicine Laboratory (No. 2025XAKJ0101006).

## Ethics Statement

Ethical approval was obtained from the Institutional Animal Care and Use Committee of Shandong University (approval number: 240142). Patients and/or the public were not involved in the design, or conduct, or reporting, or dissemination plans of this research.

## Consent

The author has nothing to report.

## Conflicts of Interest

The authors declare no conflicts of interest.

## Supporting information




**Supporting File**: advs76696‐sup‐0001‐SuppMat.pdf.

## Data Availability

The data presented in this study are available upon reasonable request from the corresponding author. All sequencing data will be uploaded and made available through managed access via the Immunological Genome Project (ImmGen, GSE107170) and GSE182159.
