# Supplementary material for: Chronic HBV Infection Disrupts CCL5‐Secreting cNK Cells and Attenuates Liver Accumulation and Activation of DCs and HBV‐Specific T Cells
Source: Adv Sci (Weinh). 2026 Jul 20;13(57):e76696. doi: 10.1002/advs.76696 (PMC13384035; doi:10.1002/advs.76696)
Supplement: Supplementary file 1 [file ADVS-13-e76696-s001.pdf]

Supplementary materials and methods to:

Chronic HBV infection disrupts CCL5-secreting cNK cells and attenuates liver accumulation and activation of DCs and HBV-specific T cells

Ailu Yang[1 #], Yucan Wang[1 #], Yating Yu[1], Jing Wu[1], Cuiping Bao[1], Zixuan Wang[1], Qiuju Han[1], Zhigang Tian[2], Jian Zhang[1]*, Huajun Zhao[1]*

[1] State Key Laboratory of Discovery and Utilization of Functional Components in Traditional Chinese Medicine, School of Pharmaceutical Sciences, Shandong University, Jinan, Shandong, 250012, China;

[2] School of Life Sciences, University of Science and Technology of China, Hefei, 230000, China

SUPPLEMENTARY MATERIALS AND METHODS

Enzyme-linked immunosorbent assay (ELISA)

ELISA kits were used to measure serum HBsAg (Autobio, Zhengzhou, China) and supernatant CCL5 (MultiSciences, Hangzhou, China) according to the manufacturers' instructions. Briefly, 50 μL of serum samples were added to the wells followed by the adding of 50 μL detection reagent. Then, plates were incubated at 37 °C for 60 min. After extensive washing, the plates were incubated with 50 μL of the chemiluminescent substrate solution (HBsAg assay) or TMB substrate (CCL5 assay) for 30 min in the dark at 37 °C. The plates were measured using the Synergy 2 Multi-Mode Microplate Reader (BioTek, Vermont, USA).

Immunohistochemistry

Tissue sections were dewaxed, rehydrated and antigen retrieved using proteinase K antigen retrieval solution (Abcam). Following this, tissue sections were washed three times with Tris-buffered saline (TBS) buffer and incubated with goat anti-Rat IgG (OriGene) as blocking reagent for 15 min. Then, intrahepatic expression of HBsAg and HBcAg were separately stained with an anti-HBsAg monoclonal antibody (#GT222429, Gene Tech Co., Ltd., Shanghai, China) and an anti-HBcAg monoclonal antibody (#GB058629, Gene Tech Co., Ltd., Shanghai, China) overnight at 4 °C. After washing with TBS buffer, tissue sections were incubated with an enzyme-conjugated goat anti-mouse/rabbit IgG polymer (#GK600505, Gene Tech Co., Ltd., Shanghai, China) and streptavidin/horseradish peroxidase conjugates (ZSGB-Bio) for 20 min at 37 °C. DAB substrate was then added followed by counterstaining with hematoxylin for analysis. Images were captured using Olympus BX46 microscope (Olympus, Tokyo, Japan).

Intrahepatic HBV DNA, HBV RNA and CCL5 mRNA detection

Intrahepatic genomic DNA was extracted via a gDNA kit (Tiangen Biotech, Beijing, China). Total hepatic RNA was extracted via TRIzol reagent (CWBIO, China) and then reverse-transcribed into cDNA via a cDNA synthesis kit (CWBIO, China). Quantitative real-time PCR analysis of intrahepatic HBV DNA, HBV RNA and CCL5 mRNA was performed via UltraSYBR reagent (CWBIO, China), as previously described [1, 2]. The

PCR primers used are shown in Supplementary Table 2.

RNA-seq analysis

Hepatic NK cells and dendritic cells from WT or HBV-carrier mice were sorted with the MojoSort™ mouse NK cell isolation kit (#480050, Biolegend) and an EasySep™ mouse CD11c positive selection kit II (#18780, STEMCELL Technologies, Canada), separately, and total RNA was extracted with TRIzol reagent (Invitrogen, Carlsbad, CA, USA). Sequencing libraries were generated via a TruSeq RNA sample preparation kit (Illumina, San Diego, CA, USA) and sequenced on the HiSeq X platform (Illumina) by Shanghai Personal Biotechnology Co. Ltd. (Shanghai, China).

Immunofluorescence analysis

Liver tissues were fixed, paraffin-embedded, and cut into 5-μm sections. After deparaffinization, rehydration and antigen retrieval, the tissue sections were preincubated with goat anti-rat IgG (#ZLI-9022, OriGene). These tissue sections were subsequently stained with anti-CD11c (#11-0114-85, eBioscience) at room temperature for at least 1 h, and CD11c$^+$ cells were subsequently analyzed with a Zeiss LSM 900 with Airyscan 2 software (Carl Zeiss, Germany).

Supplementary Table 1. Antibodies used for Flow Cytometry.

REAGENT	SOURCE	IDENTIFIER
Spark PLUS UV395™ anti-mouse/rat XCR1	Biolegend	148239
BUV615 Rat Anti-Mouse CD370 (Clec9a)	BD	752678
BUV737 anti-mouse CD11c	eBioscience	367-0114-82
BUV805 anti-mouse CD11b	eBioscience	368-0112-82
eFluor 450 anti-mouse	eBioscience	48-5698-82
eFluor 450 anti-mouse CD49b	eBioscience	48-5971-82
eFluor 450 anti-mouse lineage	eBioscience	2829434
eFluor 506 Fixable Viability Dye	eBioscience	65-0866-14
Brilliant Violet 421™ anti-mouse LAG-3	Biolegend	125221
Brilliant Violet 605™ anti-mouse CD274 (B7-H1, PD-L1)	Biolegend	124321
Brilliant Violet 605™ anti-mouse CD86	Biolegend	105036
Brilliant Violet 711™ anti-mouse IFN-γ	Biolegend	564336
BV711 Hamster Anti-Mouse CD103	BD	748255
Brilliant Violet 785™ anti-mouse TIM-3	Biolegend	119725
Brilliant Violet 785™ anti-mouse KLRG1	Biolegend	138429
Brilliant Violet 785™ anti-mouse I-A/I-E (MHC-II)	Biolegend	107645
FITC anti-mouse CD11b	eBioscience	11-0112-85
FITC anti-mouse CD11c	eBioscience	11-0114-85
FITC anti-mouse CD3e	Biolegend	100204
FITC anti-mouse NK1.1	Biolegend	108706

PE anti-mouse CD11b	eBioscience	12-0112-81
PE anti-mouse H-2Kb/H-2Db (MHC-I)	Biolegend	114608
PE anti-mouse CD69	eBioscience	12-0691-83
PE anti-mouse CD8α	eBioscience	12-0081-83
PE anti-mouse CD39	Biolegend	143803
PE H-2K(b) /VWLSVIWM Tetramer	Helixgen	HG08T21008
PE/Dazzle™ 594 anti-mouse CD8α	BD Biosciences	562283
PE eFlour 610 anti-mouse EOMES	eBioscience	61-4875-82
PerCP/Cyanine5.5 anti-mouse F4/80	eBioscience	45-4801-82
PerCP/Cyanine5.5 anti-mouse CD3e	eBioscience	45-0031-82
PerCP/Cyanine5.5 anti-mouse CD11a	Biolegend	101124
PerCP-eFluor710 anti-mouse CD27	eBioscience	46-0271-82
RB670 Rat Anti-Mouse CD195 (CCR5)	BD	770814
RY703 Rat Anti-Mouse CD172a (SIRPα)	BD	770059
PE/Cyanine7 anti-mouse CCL5	Biolegend	149105
PE/Cyanine7 anti-mouse TNF-α	eBioscience	25-7321-82
PE/Cyanine7 anti-mouse T-bet	Biolegend	644824
PE/Cyanine7 anti-mouse CD73	Biolegend	127223
PE/Cyanine7 anti-mouse CD80	Biolegend	104733
Phospho-IKBα (Ser32/ Ser36) antibody	Abmart	TP56280
AF647 anti-rabbit IgG (H+L)	SparkJade	EF0014
APC anti-mouse CD206	Biolegend	141708
APC anti-mouse CD49a	Biolegend	142606
APC anti-mouse CD127 (IL-7Rα)	Biolegend	135021

APC/Cyanine7 anti-mouse CD103	Biolegend	121432
APC/Cyanine7 anti-mouse NK1.1	Biolegend	108724
APC/Fire™ 750 anti-mouse CD191 (CCR1)	Biolegend	152511
APC anti-mouse CD11c	Biolegend	337208
AF700 anti-mouse CD8α	Biolegend	100730
AF700 anti-mouse CD45.2	Biolegend	109822

Supplementary Table 2. The RT-qPCR primers sequences.

REAGENT	Primers
Mouse-ACTB-F	5’-CATTGCTGACAGGATGCAGAAGG-3’
Mouse-ACTB-R	5’-TGCTGGAAGGTGGACAGTGAGG-3’
Mouse-CCL5-F	5’-CCTGCTGCTTTGCCTACCTCTC-3’
Mouse-CCL5-R	5’-ACACACTTGGCGGTTCCTTCGA-3’
HBV-3.5kb-RNA-F	5’-AAGCCACCCAAGGCACAG-3’
HBV-3.5kb-RNA-R	5’-GAGGCGAGGGAGTTCTTCT-3’
HBV-total-RNA-F	5’-TCACCAGCACCATGCAAC-3’
HBV-total-RNA-R	5’-AAGCCACCCAAGGCACAG-3’
HBV DNA-F	5’-CACATCAGGATTCCTAGGACC-3’
HBV DNA-R	5’-GGTGAGTGATTGGAGGTTG-3’

SUPPLEMENTARY FIGURE AND FIGURE LEGENDS

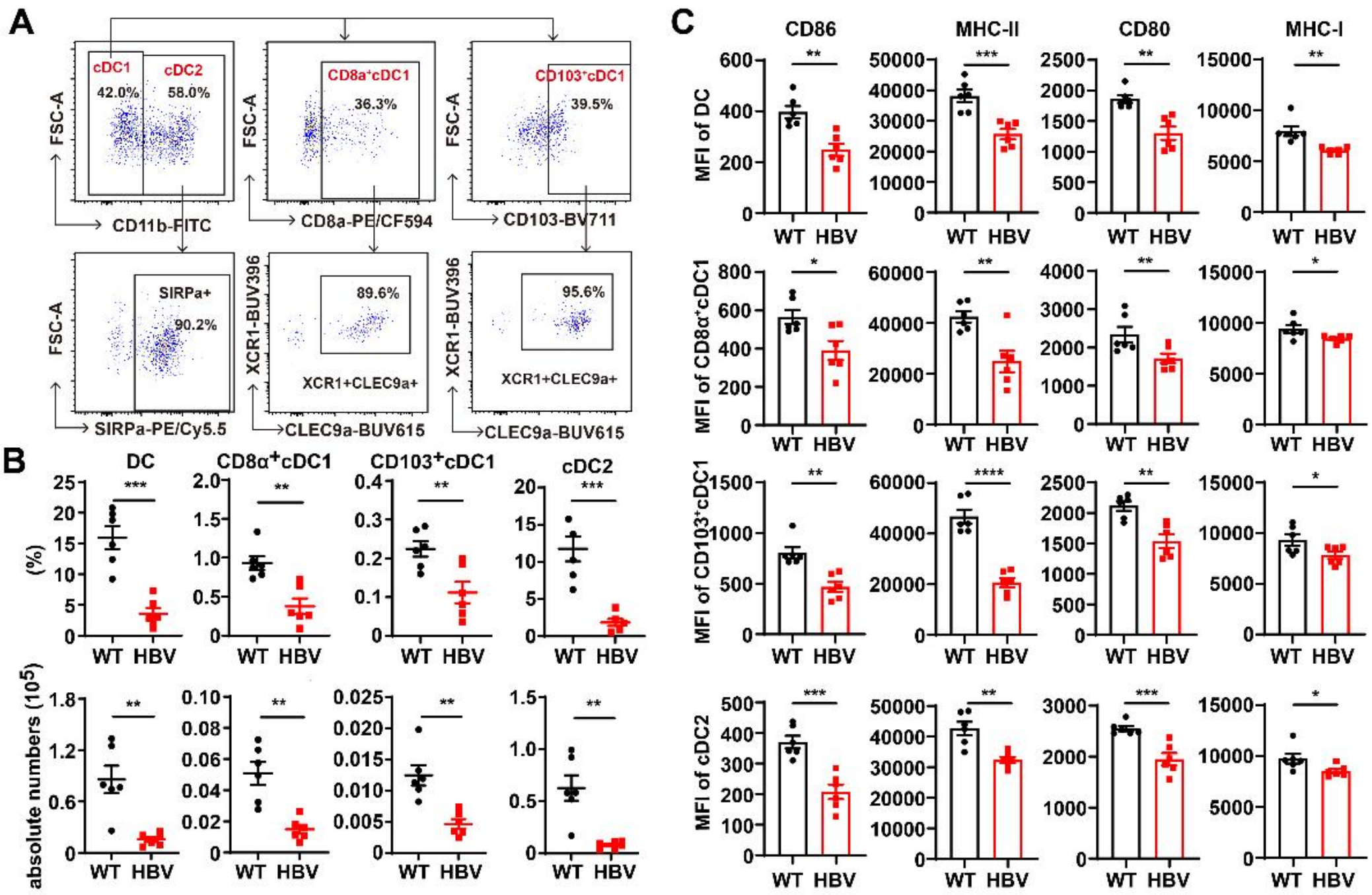

Supplementary Figure 1. Chronic HBV infection impairs the function and intrahepatic accumulation of DCs. (A-C) HBV-carrier mice were screened 6 weeks after intravenous injection of the rAAV8-HBV1.3 vector. MNCs from the liver were harvested from HBV-carrier mice. (A) The gating strategy for CD8α^{+} cDC1s, CD103^{+} cDC1s, and CD11b^{+} cDC2s in the liver. (B) The frequency and absolute numbers of DCs, CD8α^{+} cDC1s, CD103^{+} cDC1s, and cDC2s in the liver were determined by flow cytometry. (C) Flow cytometric analysis of CD86, MHC-II, CD80 and MHC-I expression on DCs, CD8α^{+} cDC1s, CD103^{+} cDC1s, and cDC2s in the liver. The data are presented as the means ± SEMs (n ≥ 5). *p < 0.05, **p < 0.01, ***p < 0.001, ****p < 0.0001.

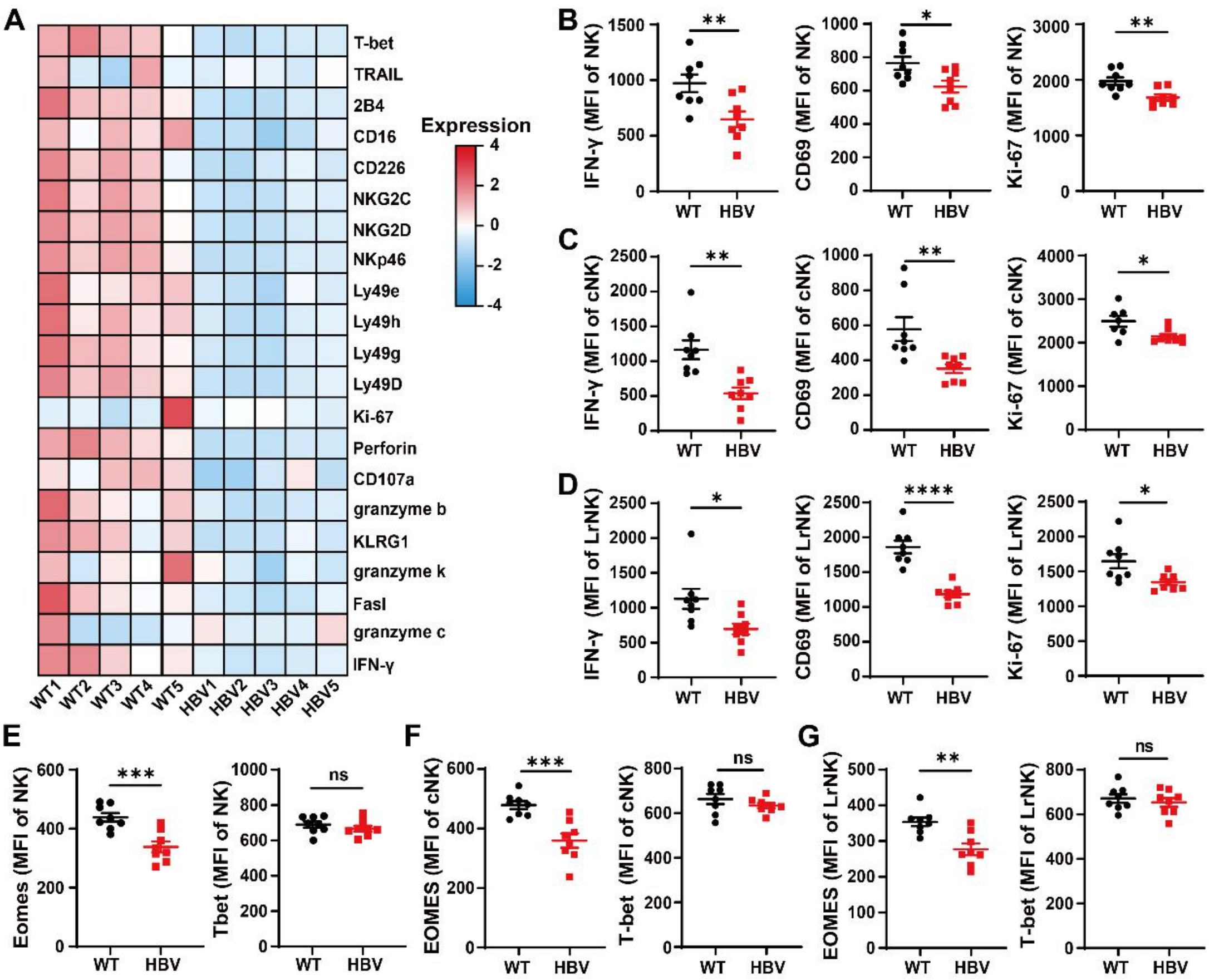

Supplementary Figure 2. Chronic HBV infection impairs the function of NK cells. (A) NK cells were harvested from the livers of WT and HBV-carrier mice, and then RNA-seq was performed. The heatmap showed the genes related to effector function and significant variation between WT and HBV-carrier mice. (B-G) Flow cytometric analysis of IFN-γ, CD69, Ki-67 (B-D), EOMES and T-bet (E-G) expression in hepatic NK cells, cNK cells and LrNK cells from WT and HBV-carrier mice. The data are presented as the means ± SEMs ($n \geq 6$). ns, not significant. $*p < 0.05$, $**p < 0.01$, $***p < 0.001$, $****p < 0.0001$.

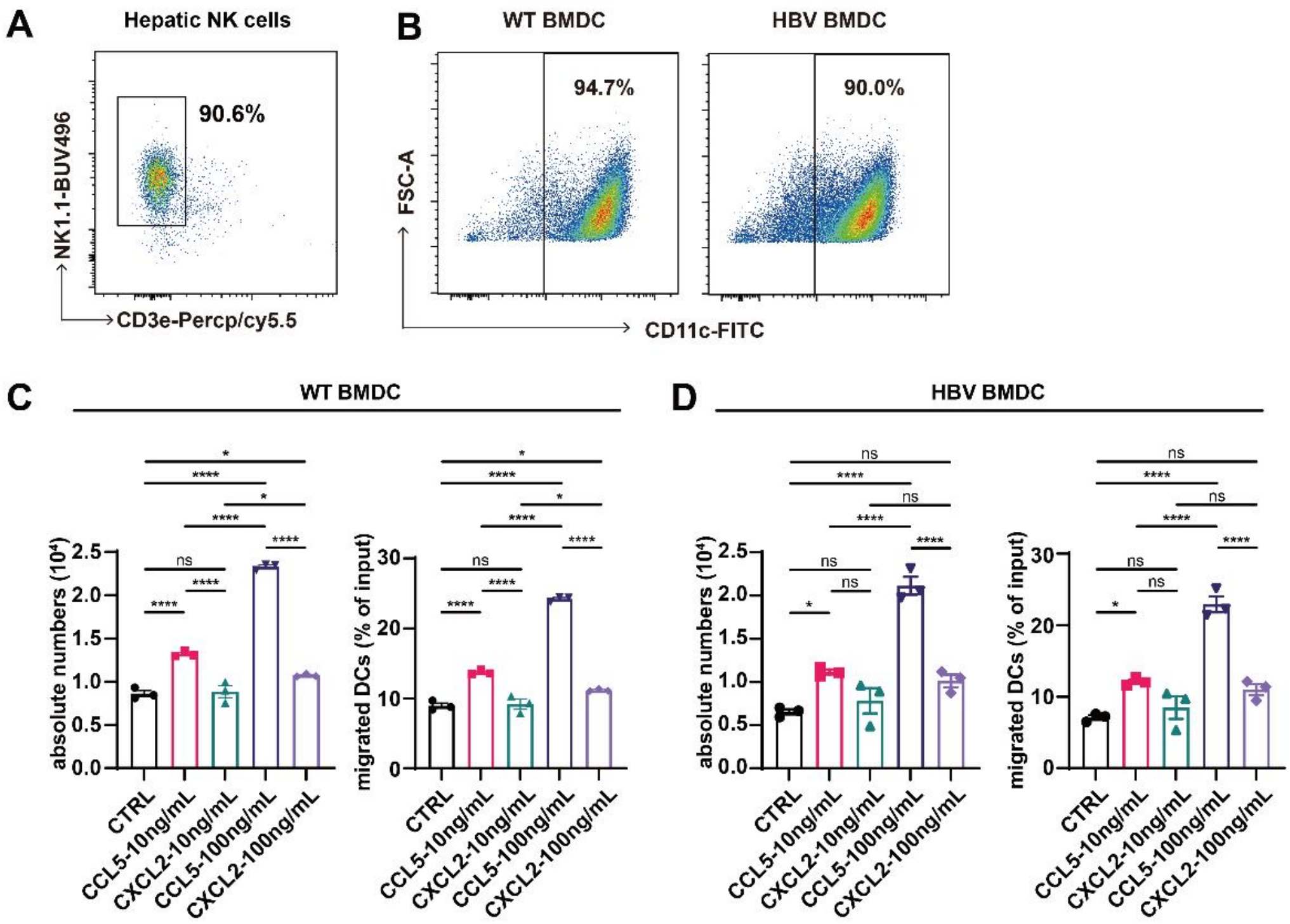

Supplementary Figure 3. Comparison of CXCL2, and CCL5 in chemotaxis of BMDCs. (A) The purity of hepatic NK cells was determined via flow cytometry. (B) The purity of BMDCs from WT or HBV-carrier mice were determined via flow cytometry. (C-D) BMDCs from WT or HBV-carrier mice were placed in the upper chamber, and 10 or 100 ng/mL recombinant CCL5 or CXCL2 was added to the lower chamber. After 5 h, the cells that migrated into the lower chamber were collected, and the number and proportions of DCs were determined via flow cytometry. The data are presented as the means ± SEMs (n = 3). ns, not significant. **p* < 0.05, ***p* < 0.01, ****p* < 0.001, *****p* < 0.0001.

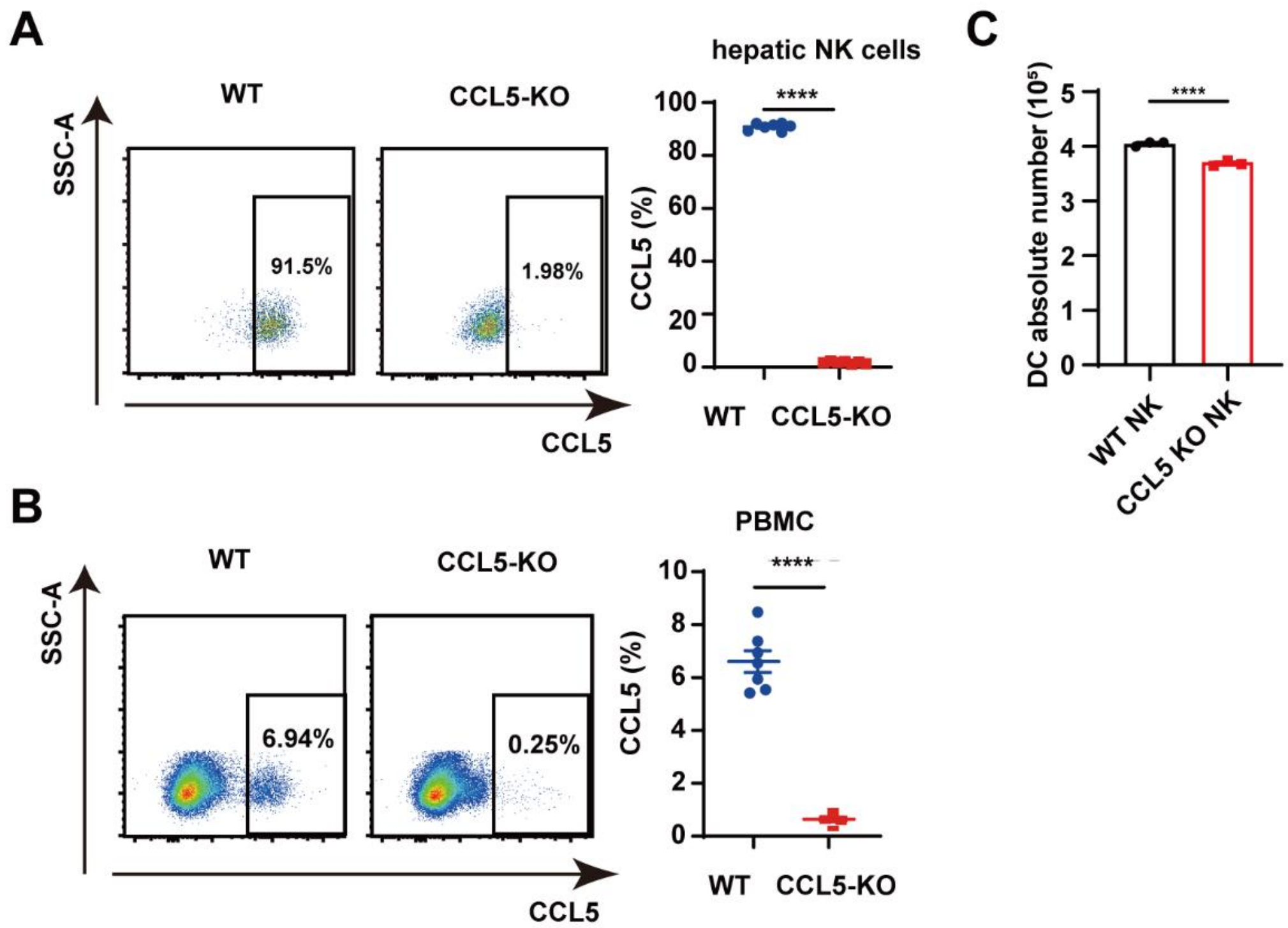

Supplementary Figure 4. The deficiency of CCL5 dampened NK cell-mediated chemotactic effects on BMDCs. The expression levels of CCL5 in NK cells in the liver (A) and PBMCs (B) from WT and CCL5-KO mice without HBV infection were analyzed via flow cytometry. (C) Hepatic NK cells were sorted from WT and CCL5-KO mice via the MojoSort™ Mouse NK Cell Isolation Kit. Then, BMDCs from HBV-carrier mice were placed in the upper chamber, and WT NK or CCL5-KO NK cells were placed in the lower chamber. After 18 h, the cells that migrated into the lower chamber were collected, and the numbers of DCs were determined via flow cytometry. The data are presented as the means ± SEMs (n ≥3). ns, not significant. *$p < 0.05$, **$p < 0.01$, ***$p < 0.001$, ****$p < 0.0001$.

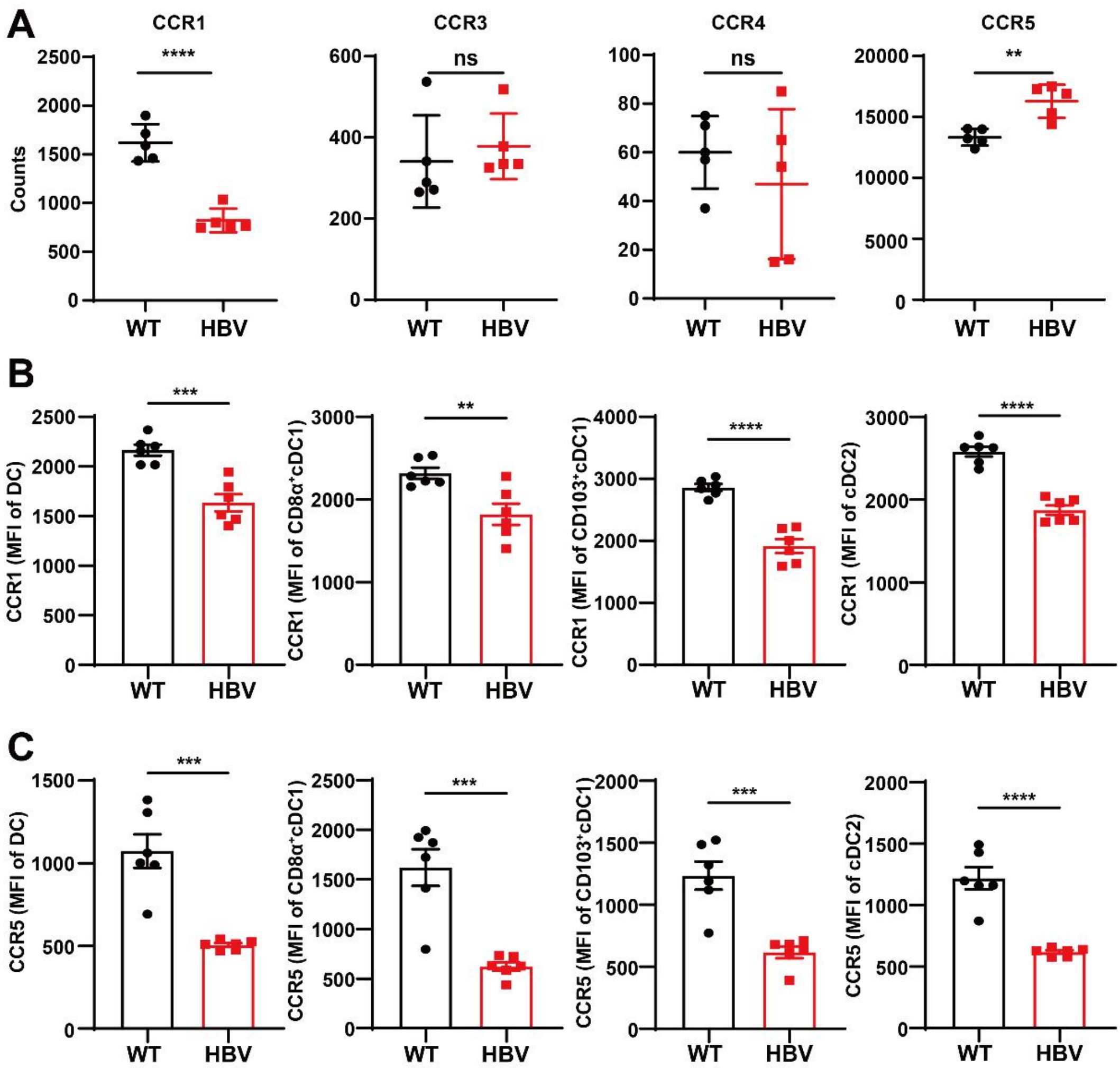

Supplementary Figure 5. The expression of CCL5-associated principal receptors on hepatic DCs. (A). DCs were harvested from the livers of WT mice and HBV-carrier mice and RNA-seq was performed. The levels of receptors on hepatic DCs were determined via RNA-seq. (B-C). Flow cytometric analysis of CCR1 (C) and CCR5 (D) expression on DCs, CD8α$^+$ cDC1s, CD103$^+$ cDC1s, and cDC2s in the liver from WT and HBV-carrier mice. The data represent the means ± SEMs (n ≥ 5). ns, not significant. *$p < 0.05$, **$p < 0.01$, ***$p < 0.001$, ****$p < 0.0001$.

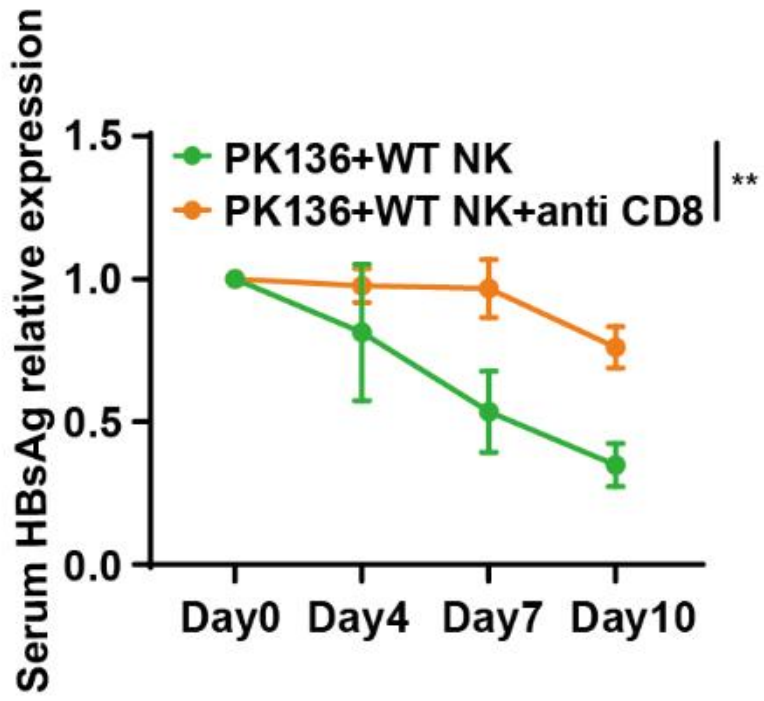

Supplementary Figure 6. Adoptive transfer of WT NK cells-induced anti-HBV effects dependent on the CD8$^+$ T cells. HBV-carrier mice were treated with anti-PK136 to deplete NK cells, and then intravenously administered 5×10^5 WT NK cells combined with or without anti-CD8 antibody. Serum was collected at the indicated time points, and the relative levels of serum HBsAg were determined by CLIA. The data are presented as the means ± SEMs (n =4). ns, not significant. **p* < 0.05, ***p* < 0.01, ****p* < 0.001, *****p* < 0.0001.

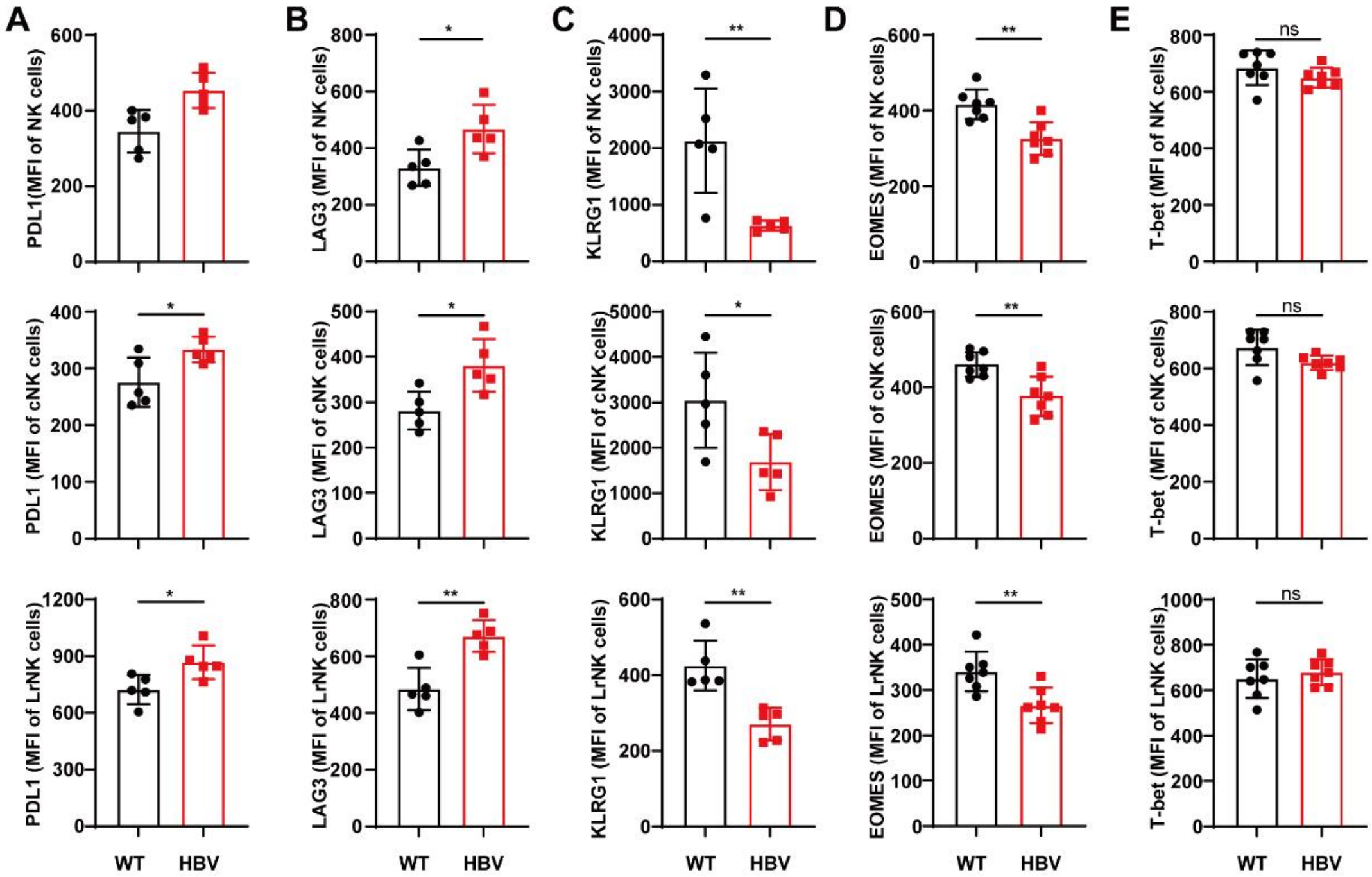

Supplementary Figure 7. Chronic HBV infection drives NK cell exhaustion and dampens differentiation. (A-F) Flow cytometric analysis of the expression levels of PDL1 (A), LAG3 (B), KLRG1 (C), EOMES (D) and T-bet (E) on hepatic NK cells, cNK cells or LrNK cells from WT and HBV-carrier mice. The data are presented as the means ± SEMs (n ≥ 5). ns, not significant. *$p < 0.05$, **$p < 0.01$, ***$p < 0.001$, ****$p < 0.0001$.

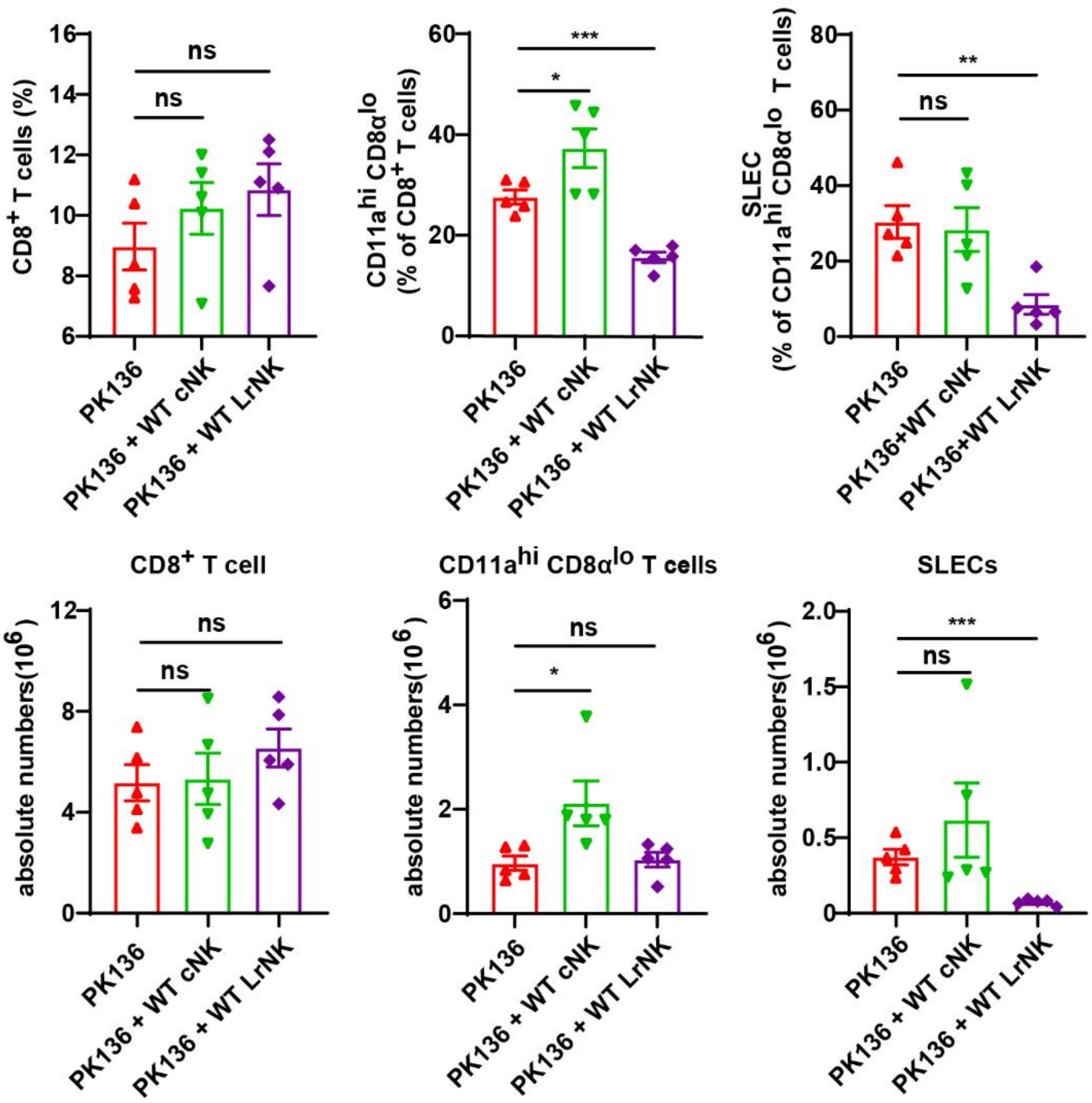

Supplementary Figure 8. cNK cells promote the proliferation and differentiation of CD8⁺ T cells in the spleen. The cNK cells and LrNK cells were sorted from the livers of WT mice via a Moflo Astrios EQ. HBV-carrier mice were treated with anti-NK1.1 to deplete NK cells and then intravenously administered 5×10^4 cNK and LrNK cells, separately. These mice were sacrificed 12 days after transfer, and the frequencies and numbers of $CD8^+$ T cells, HBV-specific $CD8^+$ T cells, and SLECs in the spleen were determined via flow cytometry. The data are presented as the means ± SEMs (n ≥ 4). ns, not significant. **p* < 0.05, ***p* < 0.01, ****p* < 0.001, *****p* < 0.0001.

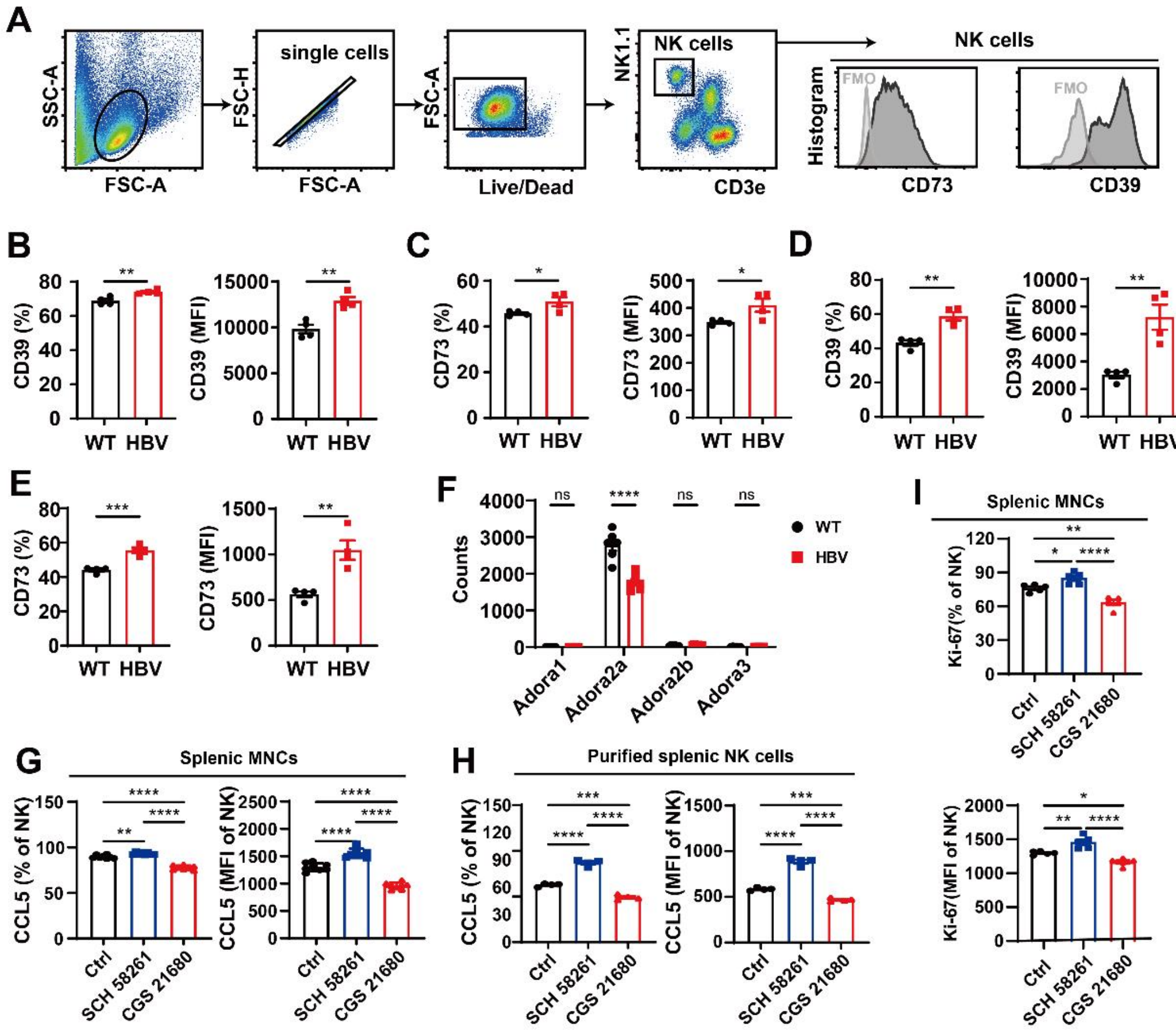

Supplementary Figure 9. Chronic HBV infection affects adenosine signaling in NK cells. (A) Gating strategy for flow cytometric analysis of CD39 and CD73 on NK cells. (B-C) Flow cytometric analysis of CD39 (B) and CD73 (C) expression on hepatic MNCs from WT mice and HBV-carrier mice. (D-E) Flow cytometric analysis of CD39 (B) and CD73 (C) expression on hepatic NK cells from WT mice and HBV-carrier mice. (F) RNA-seq analysis of adenosine receptor mRNA levels in hepatic NK cells sorted from WT mice and HBV-carrier mice. (G) Splenic MNCs from WT mice were treated with DMSO, SCH 58261 (1 μM) or CGS 21680 (1 μM) for 24 h, then the expression of CCL5 in NK cells was determined by flow cytometry. (H) Splenic NK cells sorted from WT mice via the MojoSort™ Mouse NK Cell Isolation Kit were treated with DMSO, SCH 58261 (1 μM), or CGS 21680 (1 μM) for 24 h, then the CCL5 level in the NK cells was determined via flow cytometry. (I) Splenic MNCs from WT mice were treated with DMSO, SCH 58261 (1 μM) or CGS 21680 (1 μM) for 24 h, then the expression ofKi-67 in NK cells was determined by flow cytometry. The data are

presented as the means ± SEMs (n ≥ 4). ns, not significant. *$p < 0.05$, **$p < 0.01$, ***$p < 0.001$, ****$p < 0.0001$.

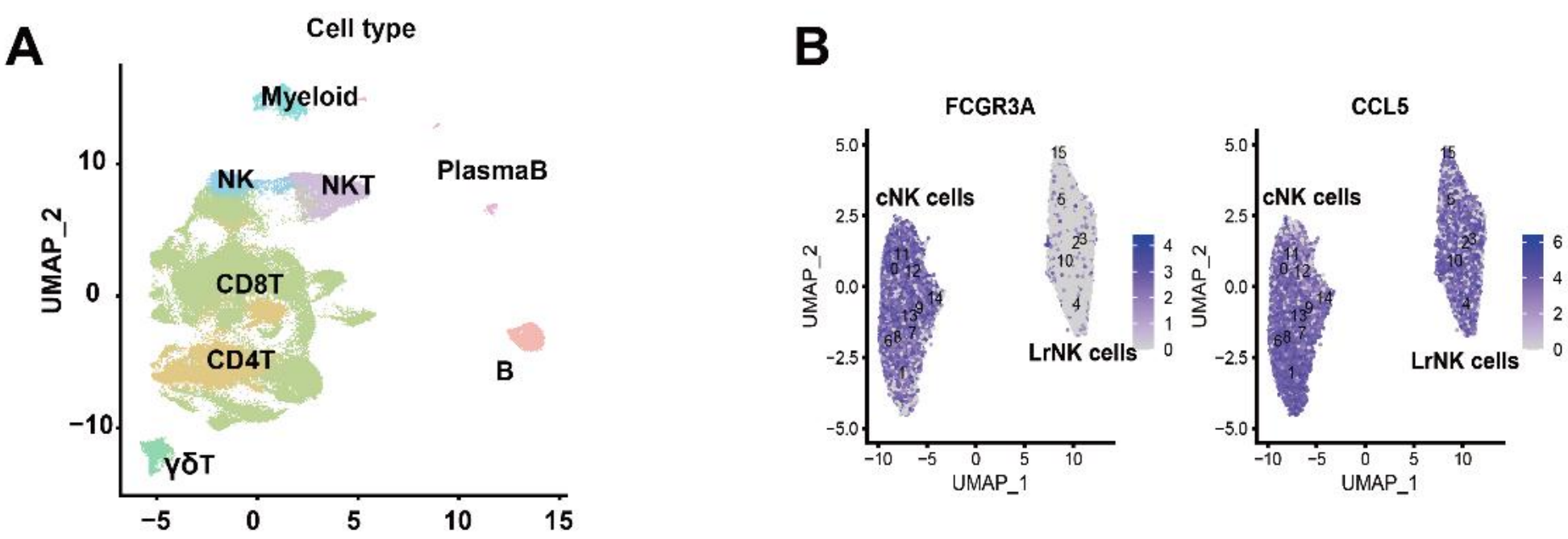

Supplementary Figure 10. Uniform manifold approximation and projection UMAP visualizations of cell subsets among CHB patients. (A) Uniform manifold approximation and projection (UMAP) visualizations of cell subsets, including hepatic NK cells, $CD4^+$ T cells, $CD8^+$ T cells, NKT cells, B cells and myeloid cells. The data were obtained from the GEO database (GSE182159). (B) UMAP plots of each marker among hepatic NK cells are shown.

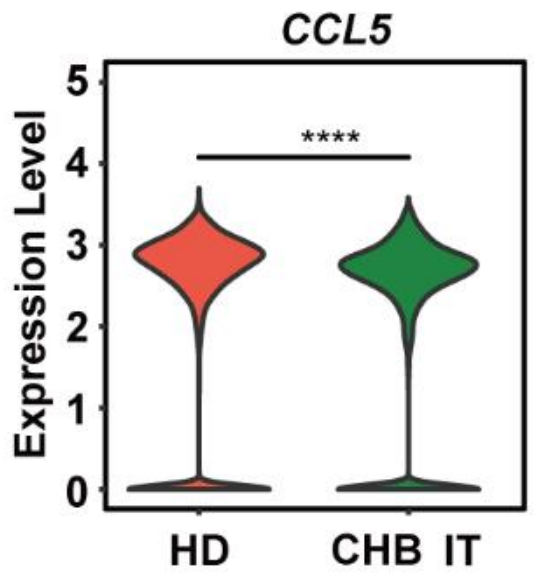

Supplementary Figure 11. The CCL5 mRNA expression of intrahepatic $CD45^+$ immune cells in the HDs and immune-tolerant (IT) CHB patients. The data are presented as the means ± SEMs ($n \geq 4$). ns, not significant. *$p < 0.05$, **$p < 0.01$, ***$p < 0.001$, ****$p < 0.0001$.

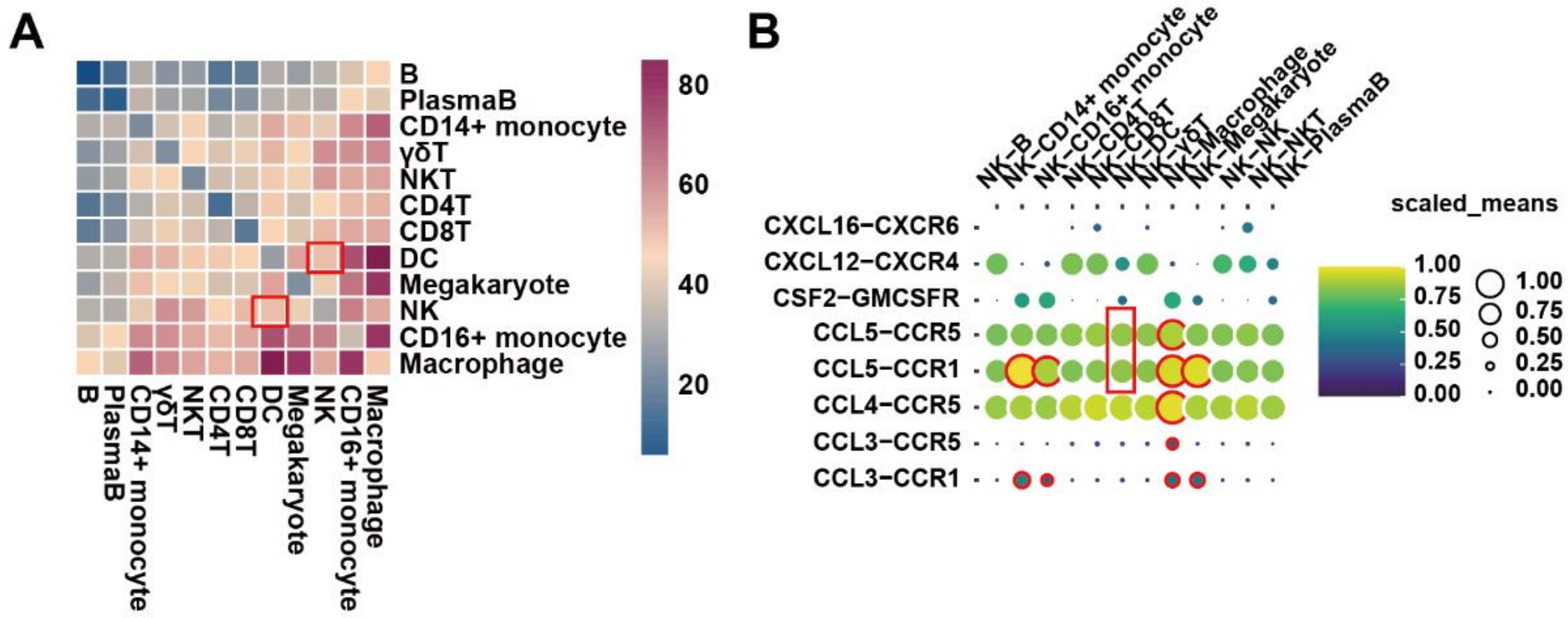

Supplementary Figure 12. CellPhoneDB-based inference of cell–cell interactions among CHB patients. (A) CellPhoneDB-based inference of cell–cell interactions between HDs and immune-tolerant CHB patients. (B) Bubble heatmap showing selected ligand–receptor pairs for interactions of NK cells and other immune cell clusters, such as $CD4^+$ T cells, $CD8^+$ T cells, NKT cells, B cells, and DCs, between HDs and immune-tolerant CHB patients. The dot size indicates the *p* value generated by the permutation test, and the color indicates the mean expression of each ligand–receptor pair.

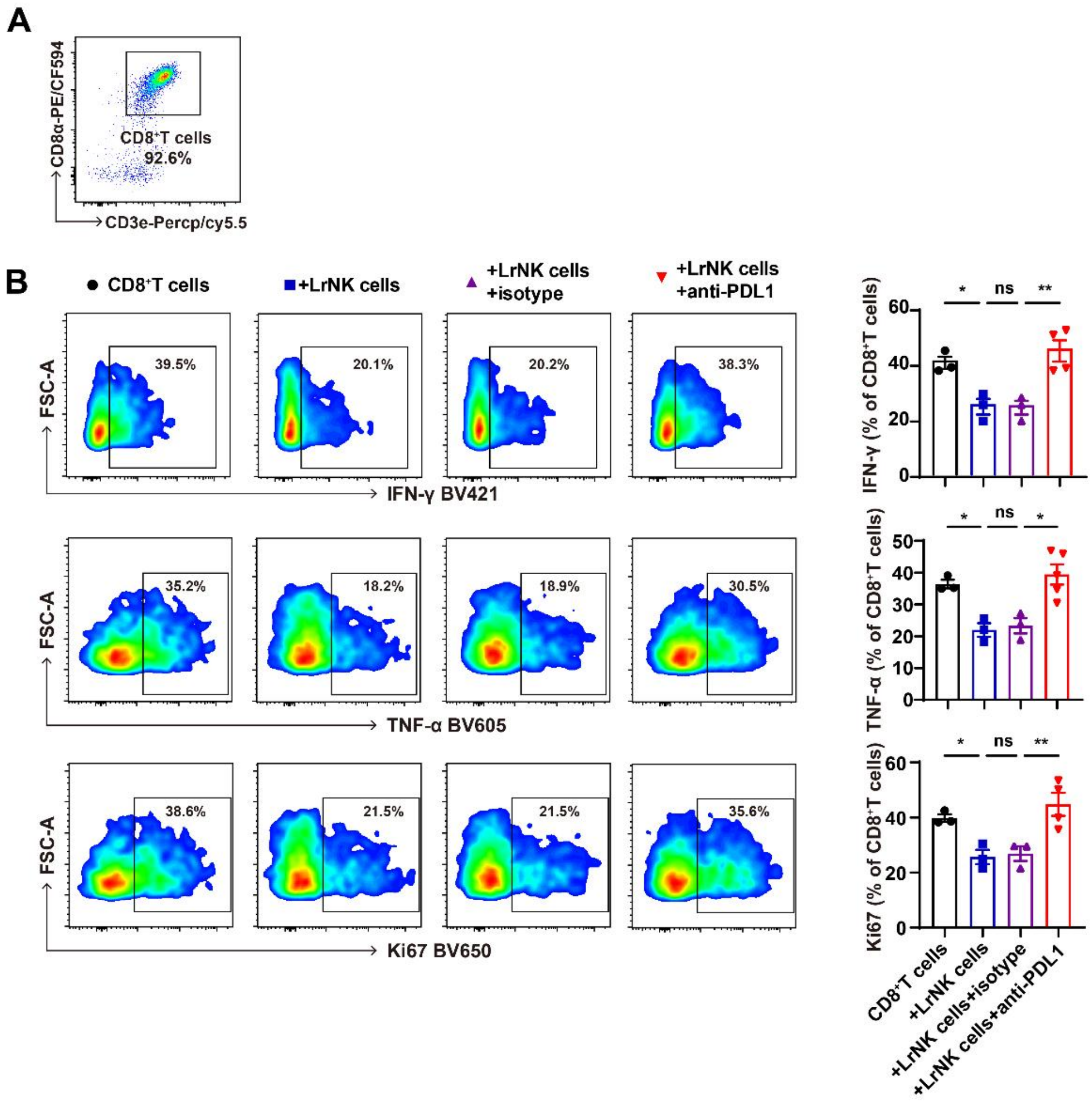

Supplementary Figure 13. LrNK cells suppress CD8⁺ T cell function through the PD-L1/PD-1 pathway. (A) Flow cytometry analysis of the purity of sorted splenic CD8⁺ T cells from HBV-carrier mice. (B) 1 × 10^5 CD8⁺ T cells were cultured alone or co-cultured with 1 × 10^5 hepatic LrNK cells from HBV-carrier mice, with or without treatment with anti-PDL1 or its isotype control. All cultures were performed in the presence of anti-mouse CD3ε (2 μg/ml) and anti-mouse CD28 (1 μg/ml). After 24 hours of culture, the expression of IFN-γ, TNF-α and Ki-67 in CD8⁺ T cells was detected by flow cytometry. The data are presented as the means ± SEMs (n ≥ 4). ns, not significant. *$p < 0.05$, **$p < 0.01$, ***$p < 0.001$, ****$p < 0.0001$.
